# A single-centre, randomised comparison of the performance and safety of a novel mechanomyography sensor with electromyography

**DOI:** 10.1016/j.bja.2025.10.067

**Published:** 2026-01-07

**Authors:** Anna S. Scholze, Bernhard Ulm, Nadine Kretsch, Bettina Jungwirth, Manfred Blobner, Flora T. Scheffenbichler

**Affiliations:** 1 Department of Anaesthesiology and Intensive Care Medicine, TUM School of Medicine and Health, Technical University of Munich, Munich, Germany; 2 Department of Anaesthesiology and Intensive Care Medicine, Faculty of Medicine, University of Ulm, Ulm, Germany; 3 Department of Anaesthesia, Intensive Care Medicine and Pain Medicine, Clinical Division of General Anaesthesia and Intensive Care Medicine, Medical University of Vienna, Vienna, Austria

**Keywords:** electromyography, mechanomyography, neuromuscular blocking agent, neuromuscular monitoring, precision

## Abstract

**Background:**

Quantitative neuromuscular monitoring is crucial to ensure patient safety when using neuromuscular blocking agents. Although historically considered as the research standard for neuromuscular monitoring, currently, no certified mechanomyography device is available for routine clinical use. This study aimed to investigate noninferiority in precision of a newly developed mechanomyography (TOF^3D^ mechanosensor; MIPM, Mammendorf, Germany) compared with electromyography.

**Methods:**

We conducted a prospective, interventional, single-centre agreement study comparing electromyography and mechanomyography in 33 anaesthetised adult patients. Devices were randomly installed on opposite arms. Train-of-four (TOF) ratios were measured beginning before administration of rocuronium 0.45 mg kg^−1^ i.v. until spontaneous recovery and after additional administration of sugammadex 2 mg kg^−1^ i.v. The primary endpoint was precision using the repeatability coefficient at baseline and full recovery. Precision of the TOF ratio measurement was assumed noninferior if the repeatability coefficient did not exceed a margin of 0.01.

**Results:**

Mechanomyograph**y** had higher repeatability coefficients than electromyography at all measured TOF intervals. Mechanomyography was partially noninferior to electromyography because the 95% confidence interval of the median difference between TOF ratios of both techniques (0.039 [0.007 to 0.042]) covered the acceptable margin during baseline, but not after recovery to TOF ratio >0.9 (0.052 [0.047 to 0.076]).

**Conclusions:**

The TOF^3D^ mechanosensor was not less precise than electromyography during baseline. Better hand fixation must be achieved to improve precision mechanomyography.

**Clinical trial registration:**

EUDAMED (CIV-23-06-043334); ClinicalTrials.gov (NCT06230653).

**Editor’s key points**

• Quantitative neuromuscular monitoring is now recommended by most guidelines to ensure patient safety when using neuromuscular blocking agents.
• Although mechanomyography is considered the research standard for neuromuscular monitoring, there is no such device available for routine clinical use. The precision of a new mechanomyography device (TOF^3D^) was therefore compared with electromyography.
• The TOF^3D^ mechanosensor was no less precise than an established electromyography device at baseline. With further development it could provide an improved alternative to current neuromuscular monitoring methods.

Quantitative neuromuscular monitoring is critical for the safe management of neuromuscular block and is now strongly recommended by both the European Society of Anaesthesiology and Intensive Care (ESAIC) and the American Society of Anesthesiologists (ASA).^1^^,^^2^ However, current standard methods such as acceleromyography and electromyography (EMG) have limitations. Acceleromyography, commonly used in clinical practice, overestimates neuromuscular recovery because of idiosyncratic train-of-four (TOF) ratios >1.0 and lacks precision and agreement with mechanomyography (MMG) and EMG.^3^^,^^4^ As the EMG measures the sum action potential, it is unaffected by mechanical interferences that can change the position of the hand and fingers. However, electrical artifacts can cause it to underestimate the depth of the neuromuscular block.^5^^,^^6^ Nevertheless, EMG is considered the ‘alternative’ gold standard in neuromuscular monitoring.^7^

Mechanomyography, which directly measures isometric muscle contraction force, is considered the research gold standard for neuromuscular monitoring.^8^ Despite this, MMG devices have not been widely used in clinical practice because of their cumbersome design and complex setup.^6^^,^^7^ Recent advances in sensor technology suggest that the usability of MMG has improved to a point where it might be routinely used clinically without loss of precision.

The TOF^3D^ (MIPM, Mammendorf, Germany) is a battery-powered, stand-alone device for quantitative neuromuscular monitoring, equipped with a three-dimensional acceleration sensor for measuring muscle response.^9^ A newly developed MMG sensor is now also available. The MMG sensor measures muscle force in one dimension using an electrically isolated force transducer integrated into a handgrip shaped to fit into the patient’s palm and secured with an adjustable elastic strap, minimising changes in sensor positioning. It can be placed on both hands even with arms tucked. The thumb is placed in a dedicated sensor tray located on the top of the handgrip which has an open design with a closable lid containing flexible elements that stabilise the thumb during measurement. The tray is slightly angled to ensure consistent contact between thumb and force transducer. According to the manufacturer's specifications, this position causes a preload in the adductor pollicis muscle of 1–3 N, depending on the size of the hand. Its maximum deflection of 30 μm at full load enables measurement of an almost isometric contraction of the adductor pollicis brevis after stimulation of the ulnar nerve at the wrist (Fig. 1).

**Fig 1:**
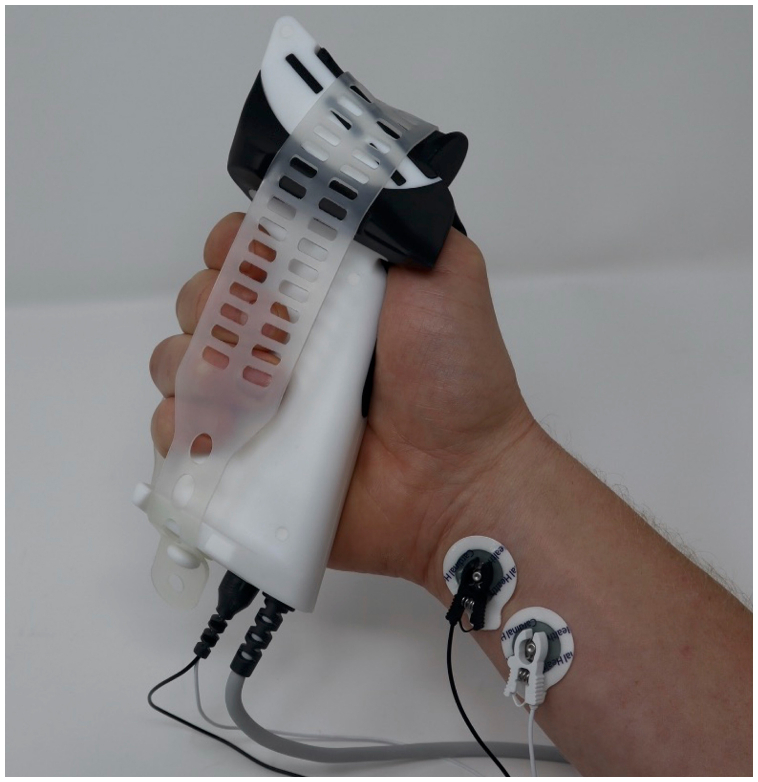
The TOF^3D^ mechanomyography sensor (MIPM, Mammendorf, Germany) consists of a handle with a force transducer and two stimulation cables. The hand is placed in the handle and fixed with a rubber band. The thumb is placed in the tray with the pressure transducer. The stimulation cables are connected and —the stimulation electrodes are attached over the ulnar nerve (further technical details can be found in the manufacturer's data sheet in Supplementary material 2).

The aim of this study was to investigate noninferiority in precision of the TOF^3D^ mechanosensor compared with an established EMG device. The TOF ratio was measured simultaneously with the TOF^3D^ MMG, and simultaneously a certified EMG device (NMT electrosensor, GE Healthcare, Helsinki, Finland) on opposite hands. Precision was compared using repeatability coefficients of both methods before induction and after complete recovery from neuromuscular block with rocuronium (primary endpoint). Secondary endpoints were the repeatability coefficients during recovery from the neuromuscular block and the agreement between both methods.

## Methods

We performed a prospective, interventional, single-centre agreement study at a university hospital in Germany which was approved by the local ethics committee (Technical University Munich, 2023-378_2-Mf-MDR-KK) and prospectively registered on ClinicalTrials.gov (NCT06230653, registered January 19, 2024). The study was registered in the European database on medical devices registry under CIV-23-06-043334. The Guidelines for Reporting Reliability and Agreement Studies (GRRAS) were followed where applicable.^10^ Data were protected based on the hospital’s policy of collection and storage of data. The study results were used to apply for the European medical CE certificate (see Supplementary material 1).

### Participant cohort

Patients eligible for participation were informed about the study and then asked to provide their written consent. Adult patients ≥18 yr old undergoing elective noncardiac surgical procedures with an expected duration of >45 min under general anaesthesia and moderate neuromuscular block were enrolled. Additional inclusion criteria were ASA physical status <3 and absence of any neuromuscular disease, documented alcohol or drug use disorder, pregnancy, breastfeeding, or puerperium (30 days after birth). Further inclusion criteria were that patients had no allergy to rocuronium or sugammadex, no need of tracheal intubation before surgery, no indication for rapid sequence induction, no contraindication for supraglottic airway management, and had neither surgery nor nerve or muscle injuries that prevent the devices from being attached to both arms.

Patients were excluded if they withdrew consent or inclusion criteria were no longer fulfilled.

### Randomisation and blinding

Participants were randomly assigned in a 1:1 ratio to neuromuscular monitoring with MMG or EMG on the dominant arm. The other device was used on the contralateral arm. Randomisation was conducted using a list generated by a random number generator, each case of which was transferred to an opaque envelope. The study physician (ASS) opened the envelope before personally installing both monitoring devices on every patient. The MMG monitor was only visible to the study physician to ensure that clinical decisions made by the responsible anaesthesiologist were based solely on EMG monitoring. The study statistician (BU) was blinded to group allocation for analysis of the primary endpoints.

### Anaesthesia protocol

Anaesthesia was induced according to the department's standard of care. Vital signs (Carescape Monitor B450, GE Healthcare), including electrocardiography, noninvasive blood pressure measurement, and oxygen saturation, were measured upon arrival in the induction room. After preoxygenation, anaesthesia was induced and maintained with remifentanil and propofol. The airway was secured with a supraglottic airway device. Participants were ventilated under pressure control at a respiratory rate of 12 bpm. Peak inspiratory pressure was set to maintain end-tidal CO_2_ partial pressure between 4.7 and 5.3 kPa (35 and 40 mm Hg). Nasopharyngeal temperature was kept >36°C. Rocuronium was administered after neuromuscular monitoring had been established. At the end of surgery, adequate recovery from neuromuscular block was ensured by an EMG-measured TOF ratio >0.9 before anaesthesia was terminated. After surgery, participants were monitored in the postoperative recovery room according to institutional standards.

### Intervention

MMG and EMG were applied to the respective hands according to randomisation following the recommendations for good neuromuscular research practice.^11^ Ag/AgCl electrodes (NMT Electrode, Solid gel; GE Healthcare, Chicago, IL, USA) were used for all stimulation and measurement purposes. Pressure points were cushioned.

EMG is the recommended ‘alternative gold standard’ to MMG.^7^ The device used has been validated against MMG.^12^ After the skin was disinfected and dried, five Ag/AgCl electrodes were attached: two stimulation electrodes on the ulnar nerve, one ground electrode on the wrist, the recording electrode over the hypothenar muscles, and the neutral electrode on the lateral metacarpophalangeal joint of the fifth finger. Care was taken to ensure that stimulation electrodes were sufficiently distant from the measuring electrodes to minimise the influence of stimulation artifacts on measurement of the sum action potentials. In accordance with the department's standard of care, the waveform of the measurements was displayed on the monitor by default.

The MMG sensor was positioned according to the manufacturer's instructions (for detail, see Fig. 1). Two stimulation electrodes were placed over the distal ulnar nerve. Both arms were positioned at an angle of 40–80° *abducted from the body* before starting the measurement and were not moved intraoperatively.

The TOF mode of both devices was set to a measuring interval of 15 s with a pulse width of 200 μs. Both devices performed an automatic search for supramaximal stimulation current, which was then used to measure the response to a TOF stimulation as a reference, a process referred to as calibration.^11^ Tetanic stimulation was performed at 100 Hz over 5 s.

Possible manipulations of arm position were documented, and if related artifacts occurred, these TOF readings were excluded from analysis. After start of surgery, both arms were covered with warming blankets (Mistral Air™, The Surgical Company International B.V., Amersfoort, The Netherlands). Skin temperature was monitored at both wrists using the skin temperature probe integrated in the TOF^3D^ mechanosensor (MMG hand) and the TOF^3D^ acceleration sensor (EMG hand) and maintained >32°C.

### Study trajectory

**Fig 2:**
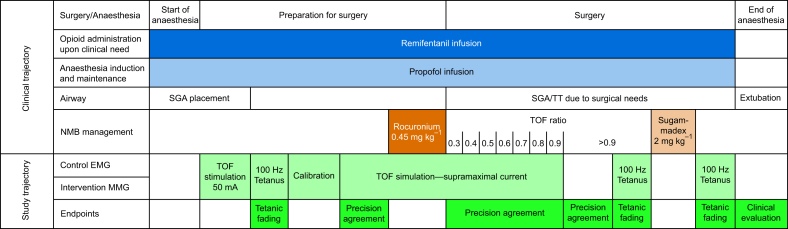
Study trajectory. MMG, mechanomyography; NMB, neuromuscular block; SGA, supraglottic airway device; TOF, train-of-four; TT, tracheal tube.

Measurements were started simultaneously using a stimulation current of 50 mA on both devices for 5 min followed by a tetanic stimulus (5 s, 100 Hz, 50 mA). After a break of 2 min, the supramaximal current was identified and used for TOF stimulation for another 5 min. Then, rocuronium 0.45 mg kg^−1^ i.v. was administered to arrive at a target TOF count <3. Additional doses of rocuronium 0.15 mg kg^−1^ were allowed to be administered until the target was achieved. The TOF ratio was monitored until it spontaneously recovered to >0.9 by EMG for 5 min. Then, a second tetanic stimulation was applied. After a 2-min break, the TOF ratio measurement was continued and sugammadex 2 mg kg^−1^ i.v. was administered. After another 5 min of TOF ratio measurement, a third tetanus was applied (Fig. 2) .

### Endpoints

The primary endpoint was precision as expressed by the repeatability coefficient at baseline and at complete neuromuscular recovery, that is, before administration of rocuronium and after neuromuscular recovery to a TOF ratio >0.9. The repeatability coefficient indicates the range within which differences between repeated measurements under unchanged conditions are expected to decrease with 95% probability. A low repeatability coefficient indicates high precision.^4^^,^^13^, ^14^, ^15^

Secondary endpoints were agreement of TOF ratios during spontaneous neuromuscular recovery and precision in seven predefined TOF ratio intervals: 0.2 to 0.3, to 0.4, to 0.5, to 0.6, to 0.7, to 0.8, and to 0.9. Further secondary endpoints relate to the handling of the MMG sensor, in particular to known problems of mechanical measurement of evoked muscle responses: incidence and magnitude of idiosyncratic TOF ratios >1.0, sensitivity of TOF ratios to changes in hand position, and deviation of final TOF ratios after recovery from rocuronium-induced neuromuscular block from baseline. Exploratory data obtained from tetanic stimulation will be reported elsewhere.

Clinical safety endpoints were respiratory and pulmonary complications in the recovery room as a possible consequence of overlooked residual neuromuscular block by both monitoring devices before removal of the laryngeal mask. Safe use of both methods was assessed by inspecting the monitored forearms and hands, looking for skin lesions, redness, and pressure points.

### Sample size

Noninferiority of precision was assumed if the repeatability coefficient of the TOF ratios of one instrument was not greater than that of the TOF ratios of the other. A difference of 0.01 between the repeatability coefficients of MMG and EMG was assumed acceptable, as it is the smallest detectable difference on commercially available quantitative neuromuscular monitoring devices displaying TOF ratios in integer percentages only. With 0.01 as the critical difference, a repeatability coefficient weighted on the published EMG data of 0.05,^4^^,^^13^^,^^15^ a significance level *P*=0.025 (sharing the two-sided alpha of 5% for the two repeatability coefficients, the one at baseline and the one at complete recovery), and a power of 80%, confirmation of noninferiority using *F*-tests needed 290 pairs of measurements. A measurement every 15 s allows 10 per patient during the 2.5-min baseline and recovery phase. With 30 patients, this results in 300 measurement pairs, which is at least the 290 required.

Bland and Altman recommend 120 pairs of measurements when repeated measurements were included in agreement studies, with the number of patients being greater than the number of repeated measurements per patient.^16^ We assumed to use at least four repetitions per participant (TOF ratio at 0.6, 0.7, 0.8, and 0.9). Therefore, the sample size of 30 participants calculated for the primary endpoint also provided sufficient power for statements of agreement. We assumed a drop-out of up to 15%, which results in five additional subjects to be enrolled. To ensure adequate training of study personnel and adherence to the study protocol, five roll-in patients were performed but not analysed.

### Statistical analysis

Precision was analysed using one-way analysis of variance on sets of consecutive TOF ratios measured by the same device. The repeatability coefficient is a measure for precision that indicates the range in which the absolute difference between two repeated test results is likely to decrease with 95% probability and has been used for describing neuromuscular monitoring devices.^4^^,^^13^, ^14^, ^15^ The 95% repeatability coefficient was calculated as $1.96\sqrt{2}{\hat{\sigma }}_{w}$, where ${\hat{\sigma }}_{w}$ is defined as the standard deviation of analysis of variance residuals.

The primary analyses had to test the noninferiority of the repeatability coefficients of the MMG-measured TOF ratios compared with those measured using EMG at baseline and at complete recovery (EMG-measured TOF ratio >0.9). A margin of 0.01 was defined as acceptable. The predefined approach for the paired samples had assumed normal distribution. The Shapiro–Wilk test, however, indicated a violation of the normality assumption. To account for the missing normal distribution and the paired study design, the 95% confidence intervals were calculated using bootstrap resampling with 2000 iterations.^17^ A method was considered noninferior if the lower limit of the confidence interval for the effect difference did not exceed the predefined noninferiority limit.

This approach was also used for the secondary analyses, the comparison of the repeatability coefficients between the two monitoring methods for the other predefined TOF ratio ranges during neuromuscular recovery.

Agreement was analysed using Bland–Altman analysis for repeated measures.^18^ The limits of agreement are calculated as 1.96 ${\hat{\sigma }}_{D}$, where ${\hat{\sigma }}_{D}$ is defined as the standard deviation of the difference between devices.

To be certain that a TOF ratio has exceeded 0.9, repetitions of the measurement are recommended. Assuming the repeated TOF readings are normally distributed, their mean value ($\bar{TOF\;ratio}$) must meet the following condition to achieve 95% confidence: $\bar{TOF\;ratio}>0.9+1.96\frac{\sigma_{i}}{\sqrt{n_{i}}}$ where *n*_*i*_ is defined as the number of TOF readings after TOF ratio >0.9 of an individual participant and $\sigma_{i}$ as standard deviation of the repetitive TOF readings. As an exploratory outcome, the number of TOF readings until the certainty of 95% TOF ratio >0.9 is reached was calculated using $\sigma_{i}$ of the TOF ratio ≥0.8 as the starting point.^19^

All analyses were performed using the statistical software R (version 4.3.3, R Foundation for Statistical Computing, Vienna, Austria).

## Results

**Fig 3:**
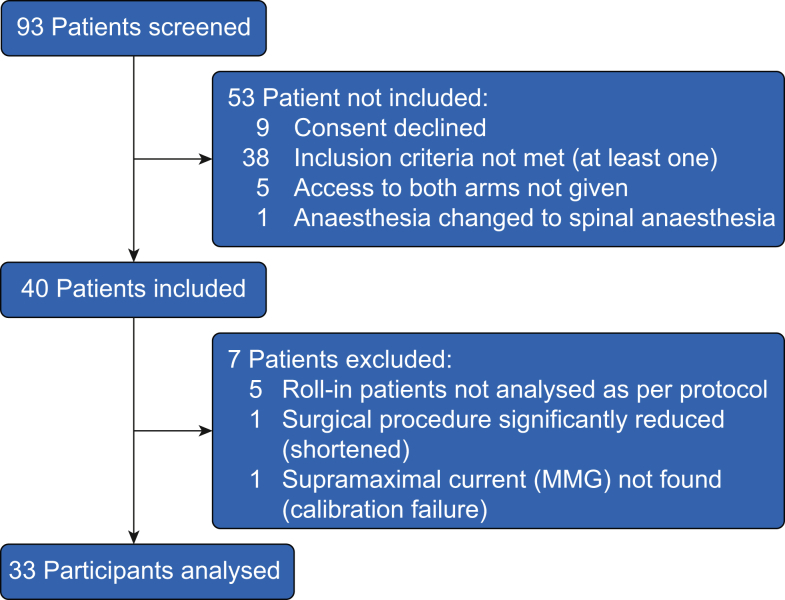
STROBE diagram. MMG, mechanomyography.

A total of 93 patients were screened between February 1, 2024, and July 17, 2024, of which 40 participants were enrolled in the study. Besides the five roll-in patients, two other patients had to be excluded from the analyses. In one patient, the study protocol had to be cancelled because of a significantly shorter operation time than planned. In another patient, a major protocol violation resulted in exclusion from analyses because a supramaximal current for the MMG device could not be found (Fig. 3).

There were five minor protocol deviations in the analysed cohort. In one participant, sugammadex was administered before spontaneous recovery of the TOF ratio >0.9 because of delayed recovery from neuromuscular block. Documentation errors occurred in four other participants: removal of the supraglottic airway device at the end of anaesthesia was not documented in three participants, and the rocuronium dose was not documented in one participant.

The study cohort comprised 33 participants with a mean age of 33 (range: 18–63) yr, 19 participants (58%) were categorised as ASA physical status 1, 10 were female (30%), and the average BMI was 25.2 kg m^−2^. The dominant hand was the right hand in 30 participants (91%) (Table 1). All participants underwent minor sport orthopaedic surgery of the lower extremity.

**Table 1:** Patient characteristics. Values are median (interquartile range) or *n* (%) of patients.

Biometrics	*n*=33
Age (yr)	32 (26–40)
Female sex	10 (30)
Height (cm)	178 (170–182)
Weight (kg)	80 (70–94)
BMI (kg m^−2^)	25.4 (22.5–28.4)
Dominant right hand	30 (91)
ASA physical status	
1	19 (58)
2	14 (42)

The supramaximal stimulation current of the TOF^3D^ MMG was 47 (IQR: 39–56) mA and that of the EMG was 51 (IQR: 45–57) mA.

In 29 participants, the TOF count was <3 on both devices after administration of rocuronium 0.45 mg kg^−1^; in three participants, one increment was required to achieve a TOF count <3 on both devices. In one participant, a second increment was required to achieve a TOF count <3 in the EMG only. After the fourth twitch response reappeared, the TOF ratio in this participant's EMG initially decreased from 0.6 to 0.3 before recovering to >0.9. During this phase, the EMG waveforms showed an atypical shape. At the same time, the TOF ratio in the MMG rose steadily during neuromuscular recovery; no decline in the TOF ratio was observed after the reappearance of its fourth twitch response.

### Precision

The repeatability coefficient of the TOF^3D^ MMG was higher (i.e. less precise) than that of the EMG during all investigated periods. Based on the predefined acceptable difference of 0.01, inferiority was not obtained during baseline measurements nor at complete recovery of the TOF ratio >0.9 (Table 2).

**Table 2:**
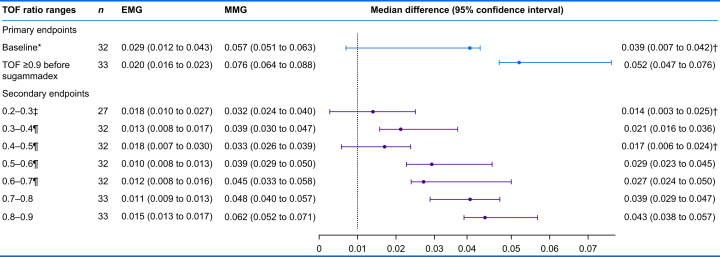
Precision expressed as repeatability coefficient. Values are median (95% confidence interval). ∗The thumb became detached from the pressure sensor during baseline. ^†^Noninferiority based on the predefined acceptable difference of 0.01, that is, the 95% confidence interval includes the margin of 0.01. ^‡^Six participants did not display an initial train-of-four (TOF) ratio between 0.2 and 0.3 on EMG or mechanomyography (MMG) during recovery. ^¶^In one participant, during recovery, the first measured TOF ratio on EMG was >0.6.

In two participants, the MMG-measured twitch responses increased by >10% from the first measurement during baseline measurements, in line with a staircase phenomenon.

During the measurements, that is, after baseline and before complete recovery to a TOF ratio >0.9, the thumb of 11 out of 33 participants slipped off the tray with the pressure transducer at least once. Two of these 11 participants required two corrections. These events were not defined as major protocol deviations, and the participants were included in the per-protocol population.

### Agreement

**Fig 4:**
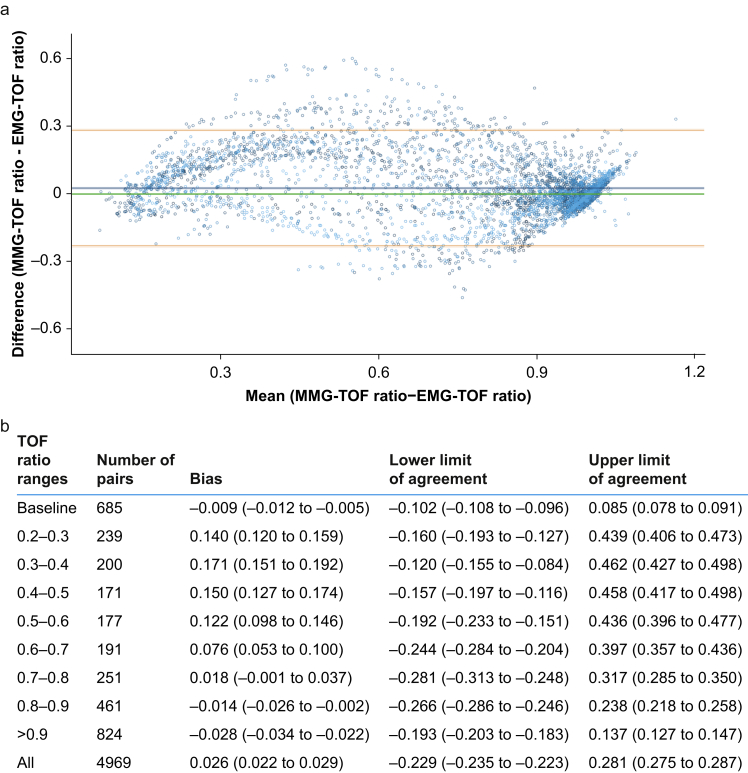
Agreement between train-of-four ratios measured using MMG and those using EMG. (a) Bland–Altman plot. (b) Table showing bias and limits of agreement. The dots of each patient have a different shade of blue. Bias and upper and lower limit of agreement are means (95% confidence interval). MMG, mechanomyography; TOF, train-of-four.

For TOF ratios <0.8, the MMG-measured higher values than the EMG (Fig. 4). In contrast, TOF ratios >0.8 were slightly lower when measured with the MMG compared with those measured with the EMG. Across all participants and measuring points, the bias between MMG and EMG was <0.03.

### Exploratory endpoints

When the TOF ratio was >0.9 for the first time, fewer repetitions were required with EMG than with MMG to achieve 95% certainty that the TOF ratio would not decrease below 0.9 again (2 [IQR: 2–2] *vs* 15 [IQR 8–15] repetitions). After administration of sugammadex 2 mg kg^−1^, EMG measurements had to be repeated once in all participants; when using the TOF^3D^ MMG, more than one repetition was necessary in 18 of 33 participants (1 [IQR: 1–13]).

### Side effects

Of 40 participants only one experienced side effects with the MMG device. After releasing the thumb from the tray and removing the handle, skin redness was observed on the dorsal distal part of the thumb. At no time did the participant express any pain in this area. At discharge to the ward no residuals were recognisable. There were no adverse safety events.

## Discussion

This agreement study revealed that the TOF^3D^ MMG is no less precise than an established EMG device during baseline measurements because the repeatability coefficient does not exceed the acceptable margin of 0.01 (median difference [95% confidence interval]: 0.039 [0.007 to 0.042], the 95% confidence interval includes the margin of 0.01). However, after recovery of the TOF ratio to >0.9 from rocuronium-induced neuromuscular block, the median difference of repeatability coefficients measured by the TOF^3D^ MMG indicated less precision compared with the EMG (0.052 [0.047 to 0.076]). We have therefore been able to meet noninferiority in one of two criteria only.

Because there is no certified MMG device available^20^^,^^21^ that can be used to evaluate the accuracy of TOF^3D^, we decided to use the most precise neuromuscular monitoring technique currently available as a benchmark, namely EMG.^4^^,^^13^^,^^15^^,^^22^^,^^23^ We confirmed the low repeatability coefficients of EMG for all TOF windows examined, with values between 0.01 and 0.03. Given the high precision of the EMG and the lowest measurable TOF ratio difference of 0.01 as an acceptable margin, it was expectedly challenging to prove noninferiority of the new TOF^3D^ MMG. Accordingly, the noninferiority of TOF^3D^ MMG compared with EMG at baseline is remarkable for a mechanical measurement technique. Nevertheless, after complete recovery of the TOF ratio to >0.9, MMG was less precise than EMG.

Previous comparisons between EMG and MMG were conducted under strict requirements for constant preload and a stable measurement geometry.^12^ These complex and rarely achievable conditions contributed to its failed establishment in clinical routine. Because the TOF^3D^ mechanosensor aims to make the MMG clinically usable, preload and measurement geometry were integrated into the design of the handgrip. However, the TOF^3D^ mechanosensor does not control the preload, meaning that the anaesthesia provider does not know if the postulated preload of 1–3 N is ever present.

The adjustable elastic strap did not completely prevent the hand from moving, nor did the lid with its flexible elements sufficiently stabilise the thumb on the sensor tray. In this study, the thumb had to be realigned on the sensor tray in a third of the participants. In this context, the ability to monitor preload would be important, as this would enable detection of any measurement-relevant dislocation of the thumb. The deterioration in precision between baseline and recovery to TOF ratio >0.9 might be explained by this instability of the measurement geometry and preload during the course of monitoring.

This study confirmed the well-known agreement between EMG and MMG for the TOF^3D^ MMG, evidenced by an almost negligible bias of <0.03 after TOF ratio recovery to >0.9.^24^ The TOF^3D^ MMG neither overestimated TOF ratio recovery nor displayed idiosyncratic TOF ratios >1.1, which is a major drawback of available accelerometers.^4^^,^^9^^,^^13^

The higher the repeatability coefficient is, the greater the uncertainty that a TOF ratio >0.9 is only an outlier. To achieve greater certainty, it is therefore recommended to repeat TOF measurements or to aim for higher TOF ratios.^19^^,^^25^ After recovery to first TOF ratio >0.9, significantly fewer repetitions were necessary to achieve 95% certainty using EMG (2 [IQR: 2–2]) compared with MMG (15 [8–15]). Importantly, when giving sugammadex 2 mg kg^−1^, this uncertainty caused by the high repeatability coefficient was not compensated by the increase in the TOF ratio in all participants.

### Limitations

Our study has several limitations. Firstly, in preclinical tests, different thumb positions and force levels had no influence on measurement accuracy. In particular, the sensor tray surface reacted with comparable sensitivity to force at every point, making the sensor suitable for different thumb sizes and positions. We therefore did not define dislocation of the thumb as a major protocol violation. However, on the basis of the results, we cannot rule out that the change in position nevertheless influenced the contraction force, possibly through a change in preload. Secondly, we did not measure the TOF ratio ipsilaterally with both devices. Therefore, we cannot rule out that differences between TOF^3D^ MMG and EMG are biased by the selection of the dominant hand. However, this risk of bias was reduced by randomly assigning the devices to the dominant hand. Furthermore, Claudius and colleagues^26^ showed that TOF ratios measured with contralateral MMG do not exhibit bias. Thirdly, we used an established EMG as the reference method. EMG carries the risk of stimulation artifacts, which can manifest as falsely high TOF ratios in the early stages of neuromuscular recovery.^5^ We observed this in a participant whose EMG waveforms were also atypical and whose TOF ratio was >0.6 when the fourth twitch response reappeared.^6^ However, the primary endpoints were analysed before administration of rocuronium and after complete neuromuscular recovery, which makes the influence of stimulation artifacts extremely unlikely, as was also evident from the TOF ratio course of the patient with suspected stimulation artifacts. Fourthly, we cannot rule out that the comparison with one of the newer EMG devices and their disposable electrode arrays would have yielded different results.^13^^,^^23^^,^^27^ Finally, we included many young patients undergoing minor orthopaedic sports surgery. These patients had well-trained muscles and therefore had strong contractions that increased the risk of the thumb slipping.

### Conclusions

The TOF^3D^ mechanosensor is no less precise than an established EMG device during baseline measurements. Improvement in hand fixation is likely to contribute to better precision of TOF ratio readings throughout the entire recording. If this is achieved, TOF^3D^ MMG could provide a viable alternative to current neuromuscular monitoring methods.

## Authors’ contributions

Designed the study, wrote the second draft: MB

Data acquisition: ASS, NK

Conducted the analysis: BU, ASS, MB

Data interpretation: MB, ASS, FTS

Drafted the manuscript: ASS

Critically revised the manuscript for important intellectual content and approved the final version to be published: all authors agree to be accountable for all aspects of the work in ensuring that questions related to the accuracy or integrity of any part of the work are appropriately investigated and resolved: all authors

## Data availability statement

Because of legal requirements, we are not allowed to store data, although it is de-identified, in a publicly accessible repository. To gain access, proposals should be directed to the corresponding author. Requestors will need to sign a data access agreement.

## Funding

Mammendorfer Institut für Physik und Medizin GmbH (Mammendorf, Germany).

## Declarations of interest

MB received research support from MSD (Haar, Germany), fees for consultancy or lectures from GE Healthcare (Helsinki, Finland), Grünenthal (Aachen, Germany), Senzime (Landshut, Germany), and fees for consultancy from MIPM (Mammendorf, Germany), and HW Pharmaconsulting (Moosbach, Germany). The other authors declare that they have no conflicts of interest.
