# Supplementary material for: A single-centre, randomised comparison of the performance and safety of a novel mechanomyography sensor with electromyography
Source: Br J Anaesth. 2026 Jan 7;136(3):906–14. doi: 10.1016/j.bja.2025.10.067 (PMC12975375; doi:10.1016/j.bja.2025.10.067)
Supplement: Multimedia component 1 [file mmc1.docx]

**A single centre, randomised, prospective agreement study to determine the clinical performance and safety of a novel mechanomyography sensor compared with an electromyograph**

**Authors:**

**Anna S Scholze,^1^ Bernhard Ulm** (ORCID: 0000-0002-9396-2510),**^1,2^ Nadine Kretsch,^1^ Bettina Jungwirth** (ORCID: 0000-0001-9749-7460),**^2^ Manfred Blobner** (ORCID: 0000-0002-0370-5247),**^2,3^ Flora T Scheffenbichler (ORCID: 0000-0003-1128-0565)^2^**

**Affiliations**

**^1^ Technical University of Munich, TUM School of Medicine and Health, Department of Anaesthesiology and Intensive Care Medicine, Munich, Germany**

**^2^ University of Ulm, Faculty of Medicine, Department of Anaesthesiology and Intensive Care Medicine, Ulm, Germany**

**^3^ Medical University of Vienna, Department of Anaesthesia, Intensive Care Medicine and Pain Medicine, Clinical Division of General Anaesthesia and Intensive Care Medicine, Vienna, Austria**

**Corresponding Author:**

**Prof. Dr. Manfred Blobner
Department of Anaesthesiology and Intensive Care Medicine
Universitätsklinikum Ulm
Albert-Einstein-Alle 23
Ulm 89081, Germany**

**E-mail:** [manfred.blobner@uni-ulm.de](mailto:manfred.blobner@uni-ulm.de) **phone: +49-731-500-60436**

**Table of contents**

[Supplementary information on the CE certification process 2](#_Toc211333956)

[GRRAS statement 3](#_Toc211333957)

## Supplementary information on the CE certification process

The aim of the manufacturer of the TOF^3D^ MMG was to apply for the European medical CE certificate. Since the DIN EN IEC 80601-2-49, as amended in October 2020, does not specify criteria for devices to monitor the neuromuscular transmission function, we decided to investigate whether the TOF^3D^ MMG is no less precise than a certified EMG device. There is no predefined acceptable difference for the repeatability coefficient to demonstrate non-inferiority. As the commercially available quantitative neuromuscular monitoring devices display the TOF ratio in integer percentages we used the smallest detectable difference, specifically 0.01.

1. International Electrotechnical Commission. DIN EN IEC 80601, Medical electrical equipment, Part 2-49: Particular requirements for the basic safety and essential performance of multifunction patient monitoring equipment, German version: *VDE Verlag;* 2020.

## GRRAS statement

| **Section** | **Item #** | **Checklist item** | **Reported on page #** |
| --- | --- | --- | --- |
| **Title/Abstract** | **1** | Identify in title or abstract that interrater/intrarater reliability or agreement was investigated. | 2 |
| **Introduction** | **2** | Name and describe the diagnostic or measurement device of interest explicitly. | 3-4 |
|  | **3** | Specify the subject population of interest. | 5 |
|  | **4** | Specify the rater population of interest (if applicable). | n/a |
|  | **5** | Describe what is already known about reliability and agreement and provide a rationale for the study (if applicable). | n/a |
| **Methods** | **6** | Explain how the sample size was chosen. State the determined number of raters, subjects/objects, and replicate observations. | 8 |
|  | **7** | Describe the sampling method. | 8 |
|  | **8** | Describe the measurement/rating process (e.g. time interval between repeated measurements, availability of clinical information, blinding). | 7 |
|  | **9** | State whether measurements/ratings were conducted independently. | 5 |
|  | **10** | Describe the statistical analysis. | 9 |
| **Results** | **11** | State the actual number of raters and subjects/objects which were included and the number of replicate observations which were conducted. | 10 |
|  | **12** | Describe sample characteristics of raters and subjects (e.g. training, experience). | 10 |
|  | **13** | Report estimates of reliability and agreement including measures of statistical uncertainty. | 10-11 |
| **Discussion** | **14** | Discuss the practical relevance of results. | 12-13 |
| **Auxiliary material** | **15** | Provide detailed results if possible (e.g. online). | n/a |
