# Supplementary material for: A single-centre, randomised comparison of the performance and safety of a novel mechanomyography sensor with electromyography
Source: Br J Anaesth. 2026 Jan 7;136(3):906–14. doi: 10.1016/j.bja.2025.10.067 (PMC12975375; doi:10.1016/j.bja.2025.10.067)
Supplement: Multimedia component 2 [file mmc2.pdf]

TOF3D Monitor

User Manual

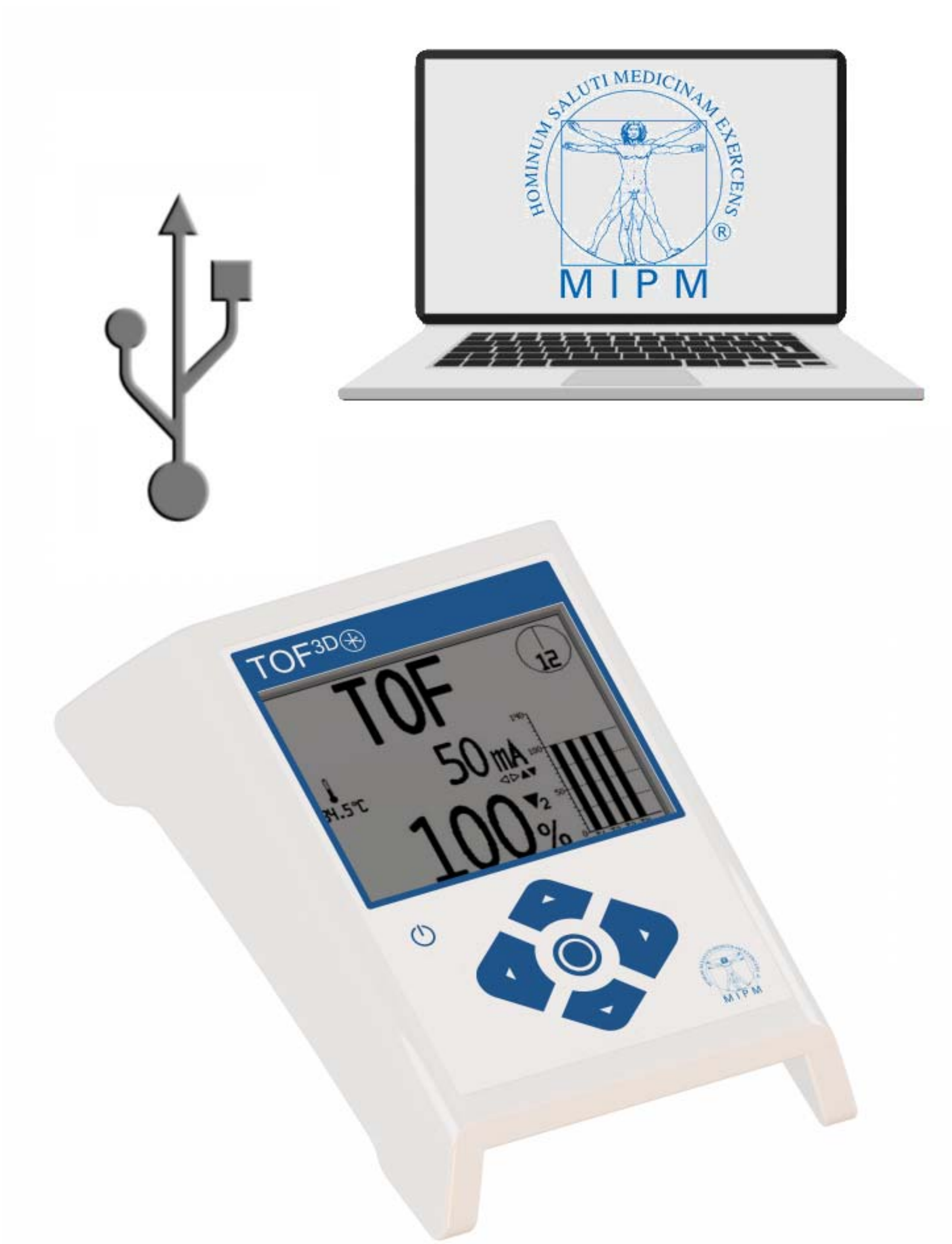

Version 3.0

Contents

General information 3
Getting started 4
File Window 9
File Menu 14
File|New file 15
File|Open File....17
File|Save File 20
File|Save_As....21
File|Export File 23
File|File info....24
File|Print All 27
File|Print info window 28
File|Print graphics window 29
File|Logout 30
Edit Menu....31
Search Menu....31
Options Menu....32
Options|Environment....33
Options|File window 35
Options|File window|Info window 36
Options|File window|Graphics window....38
Options|Legend Window 40
Options|Status Bar 41
Options|Standard Comments....43
Options|Alarm guides....44
Options|Users 46
Window Menu 48
Help Menu....48
PopUp Menu 48
Glossary....49
Index 50

General information

Welcome to the TOF3D Monitor general help!

The TOF3D presents all relevant data (twitches, body temperature, sensor baseline, stimulation mode, etc.) immediately and clearly on its LCD display. The TOF3D Monitor program is an application program which makes the real time data recorded by the TOF3D accessible for further processing on a personal computer.

The TOF3D Monitor is a "*non-clinical use*" PC software that shall not be used for active medical applications. It is intended for training and study purposes only. In case of infringement we, as the manufacturer, assume no liability for the use of the software.

The three basic functions of the TOF3D Monitor are:

1/
To record and to display recorded data coming from the TOF3D on the personal computer.

2/

To remote control the TOF3D device.

3/
To display, export and print previously recorded files stored on a personal computer.

Menu commands and Tool bar

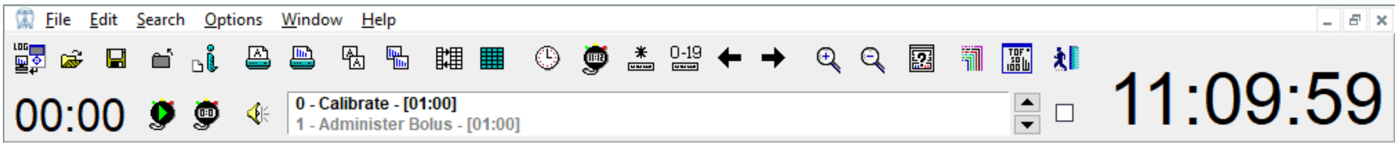

Click on the menus or on the toolbar to get information on the various functions of the TOF3D Monitor program or click Getting Started to see an example of how to use the program.

Getting started

This is an introduction where you will be asked to perform some easy tasks in order to become familiar with the basic features of the TOF3D Monitor program.

The following procedures instruct you to use the keys to select a field, box or button in a dialog box. Use Tab or Shift + Tab to select fields, list boxes and buttons in a dialog box. Use Enter to confirm your choices or Esc, if you want to cancel your choices or leave the dialog box. You can also use a mouse to click on fields or buttons.

Start up and log in to the program

Start the TOF3D Monitor program by clicking on the related icon in the Windows Start menu.

Before the TOF3D Monitor can be used you will have to identify yourself to the program. This is accomplished by typing your user ID followed by Enter. Now type in your password and accept to tick the "Not for clinical use" followed by Enter.

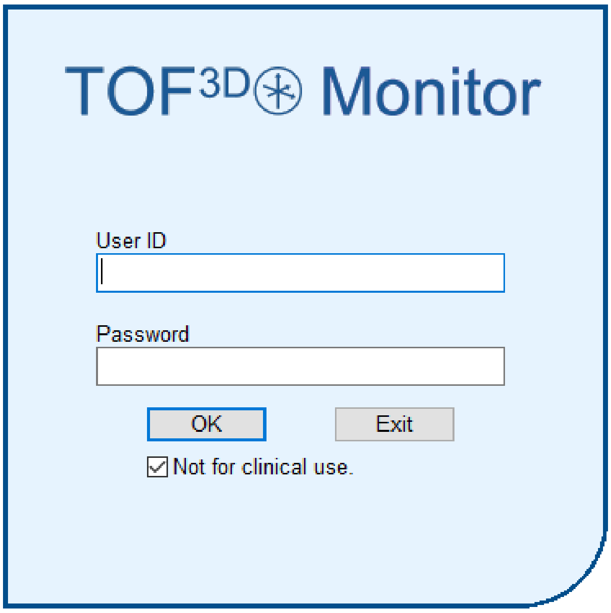

If you entered a valid combination of ID and password, the main program window becomes active. Please note that you must contact your Supervisor to get your own personal user ID and password. The Supervisor controls which kind of tasks users are allowed to perform. These tasks are:

Create and save files: You are allowed to create, modify and save files.

Open and view files: You can only view files and you are not allowed to make any changes to them.

To get full access you must be granted both of the above privileges.

Set the port options

To activate the Options|Environment dialog box press:

Alt + O (Options)

E (Environment)

The Options|Environment dialog box appears on the screen.

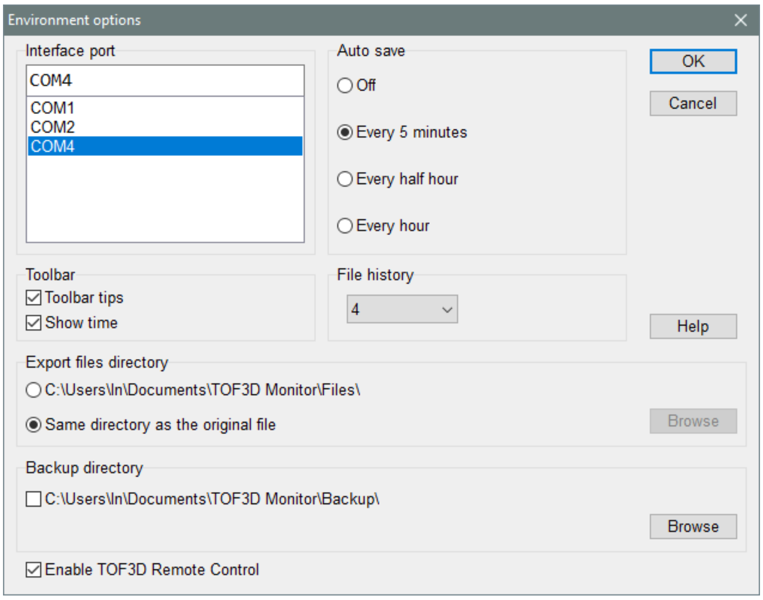

If not already done, click on the Interface port that connects to the TOF3D in the above TOF3D-LINK interface port list.

Windows will automatically assign a serial port name to TOF3D-LINK interface.

Set the Auto save to every 5 minutes to ensure minimum data loss in case of a power break down.

Press Enter to confirm your choices.

Note:

The port assignment made by Windows can be seen and modified in:

Windows Control Panel / System / Hardware / Device Manager / Ports (COM & LPT).

To change the port number, click on the Serial USB Port (COMx) / Port Settings / Advanced and change the COM Port Number.

New file

Connect the TOF3D-LINK interface to the TOF3D device and connect the TOF3D-LINK interface to a proper USB port on a PC and power On TOF3D .

To start logging recorded data click on File|New file, or press:

F2 (New file)

The File|New file dialog box appears on the screen, with the cursor in the File Name field.

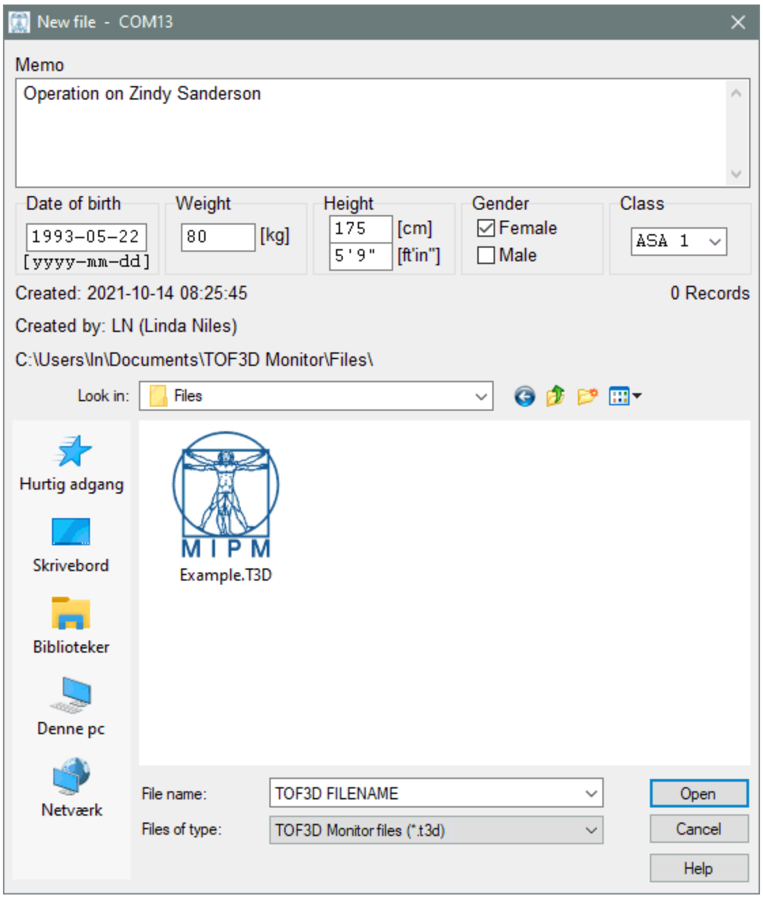

Type the name of the file you want to contain the recorded results. As an example, type:

TOF3D FILENAME

Press Tab to move to the Memo field, where you can make notes about the file. As an example, type:

Operation on Zindy Sanderson ......

If required also fill in the demographic data fields (Date of birth, Weight, Height, Gender and Class.)

Press Tab until the OK button receives focus and Press Enter.

The program will now show the File Window and try to connect to the TOF3D.

Press Esc to cancel the creation of a new file.

Press Ctrl + F4 to close the file window and end recording mode.

The Save File dialog box will appear and remind you to edit the file memo before saving the file.

Note:

If the connected TOF3D is not stimulating and contains previously recorded data a dialog will pop up asking what to do with the existing TOF3D data.

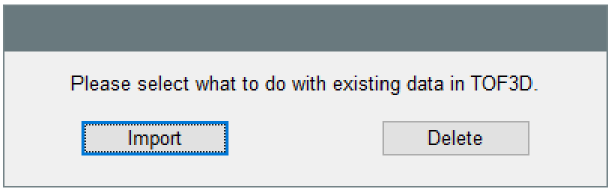

Select [Import] to import existing TOF3D data into the newly created file.

Select [Delete] to erase existing data in TOF3D and prepare the device for a new patient.

Warning:

Deleting existing TOF3D data cannot be undone.

Load and/or print a file

To activate the File|Open file dialog box, press:

F3 (Open file)

The File|Open file dialog box appears on the screen.

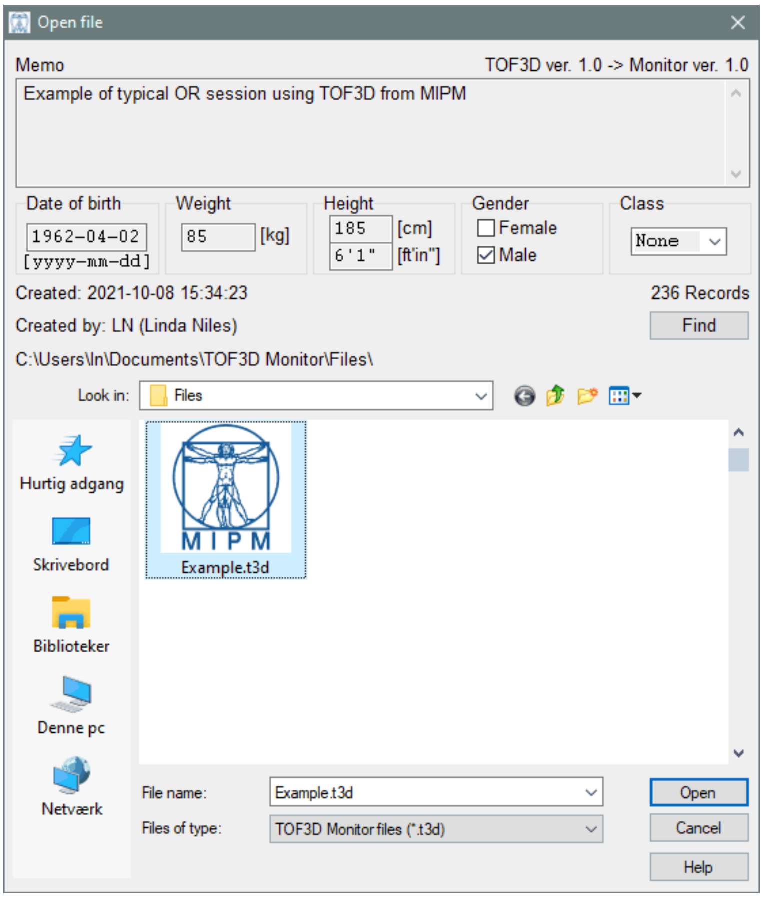

To select the file you want to display and/or print, double click on the file.

The file data will appear in a File Window. If you want to print the graph, press

Alt + F (File)

G (Print graphics window)

When the Print graphics window appears just press Enter (or click OK button) to print the current graphics window in "Fit to page" mode.

Press Ctrl + F4 to close and save the file.

Press Alt + X to exit the program.

File Window

The File Window comprises three windows: the Info, Zoom and Graphics Window. These windows can be individually toggled on/off using the Options|File Window dialog box and they can be resized by dragging the spacer lines between each window up and down.

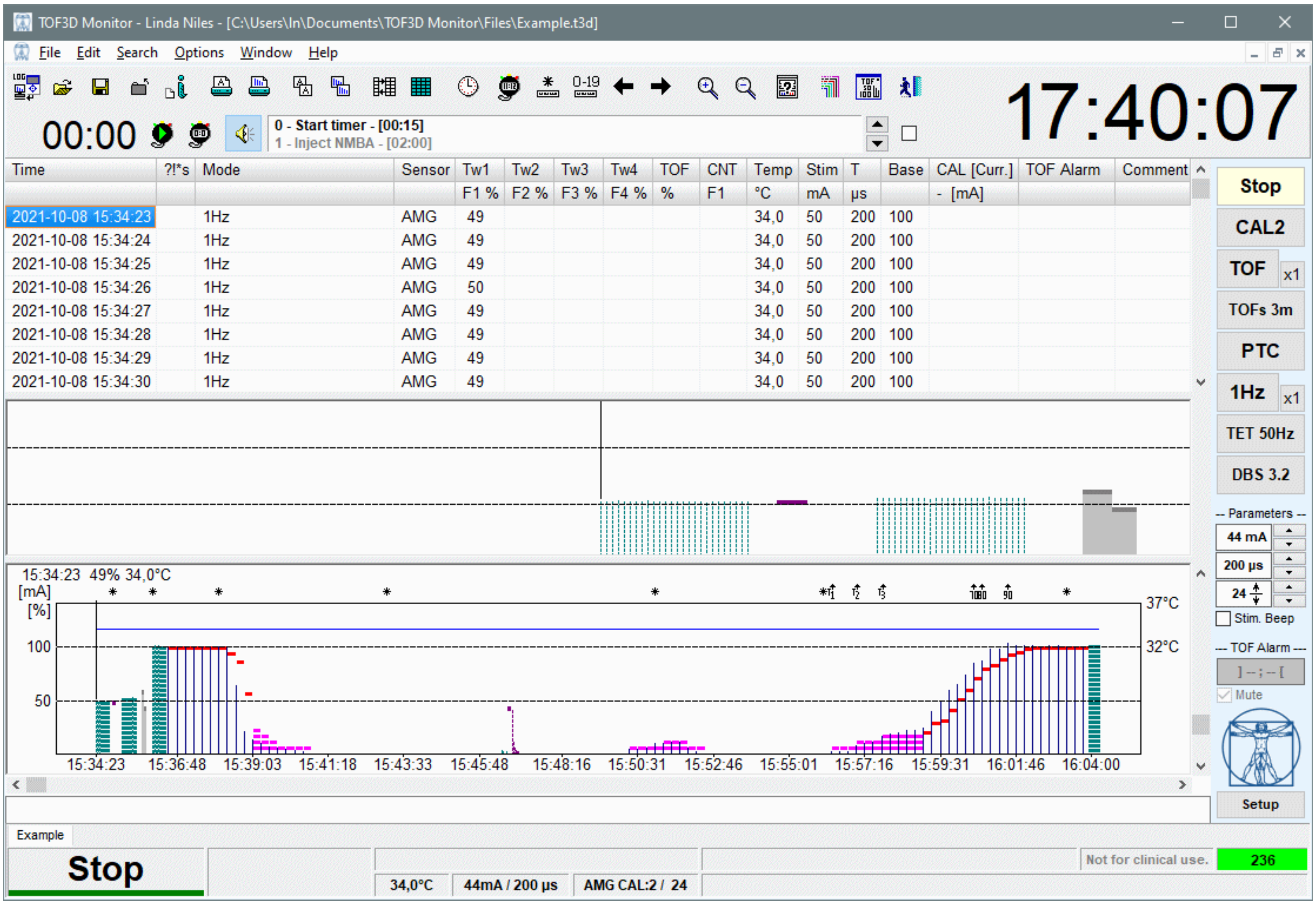

Each of the three windows has a predetermined method for presenting the TOF3D data. The windows are all correlated, (i.e. there can only be one selected event in the file to which the cursor is pointing.)

Info Window
The Info Window (upper window) contains information about the currently selected twitch (i.e., the twitch which the Twitch Cursor in the Zoom Window and the Graphics Window is pointing at). The window is built up in rows and columns like a spreadsheet. See the Options|File window dialog box for field description. Navigate in the Info Window by clicking with the mouse or using arrow, page up/down or home/end keys.

Either a part of or the whole Info Window can be selected and copied to the Clipboard. To select an area with the mouse, click on one of the corner cells in the area to be selected, hold it down and drag to the opposite diagonal corner.

To select an area with the keyboard, move the cursor to one of the corner cells in the area to be selected, press and hold down the Shift key while moving the cursor to the opposite diagonal corner with the arrow keys. Please note that the currently selected area will be marked by a gray box in the Zoom/Graphics Window.

By right-clicking anywhere in the Info window a small Pop-Up menu will appear.

Zoom Window
The Zoom Window (middle window) is a 'selective enlargement' of the twitches close to the Twitch Cursor in the Graphics Window. A twitch in the Zoom Window can be selected by clicking with the mouse directly on the twitch.

By right-clicking on the grey selection box a small Pop-Up menu will appear.

Graphics Window

The Graphics Window (lower window) shows a graph presenting a window of all twitches.

When a pre recorded file is opened, the data are compressed so that the entire session fits in the Graphics Window.

To move the Twitch Cursor from left to right or right to left, you can use a mouse or the keyboard (refer to the list below for which keys to use). If you use a mouse, just click directly on the twitch to which you want to move the Twitch Cursor. If the target twitch is not within the visible part of the file, scroll the file by dragging the thumb tab on the scroll bar below the Graphics Window or by dragging the graph itself by pressing down the right mouse button while moving the mouse. Scrolling is also possible using a mouse with a scroll wheel. The scroll bar indicates the position of the visible part of the file relative to the whole file. [Data of the twitch which the Twitch Cursor is pointing at are presented in the Info Window.]

To select an area in the Graphics Window, hold down the [Shift] key while clicking/dragging the Twitch Cursor. Please note that the selected area will also cause the related rows in the Info Window to become selected.

The Graph can be freely zoomed in and out by means of the scroll bar to the right of the Graphics Window or by means of the two zoom +/- buttons located in the tool bar. Alternatively a mouse equipped with a scroll wheel can be used. Press [Ctrl] to zoom in/out by means of a scroll wheel or press [Ctrl] + [Shift] to fine-tune the zoom factor.

Below the graph, Time is given in hours, minutes and seconds (hh:mm:ss).

By right-clicking on the grey selection box a small Pop-Up menu will appear.

Legends and symbols

Every type of stimulation is represented in the graphics window by colored vertical lines.

A red TOF ratio bar represents the calculated TOF ratio - T4/T1 [%].

The number of present pink TOF count bars represent the number of detected TOF twitches in case the TOF ratio cannot be calculated.

The elevation of a purple bar depicts the stimulation strength [mA] of a 5 sec. Tetanic stimulation used in TET & PTC.

Where no patient response has been recorded elevated small vertical dots depicts the stimulation strength [mA] for the given stimulation.

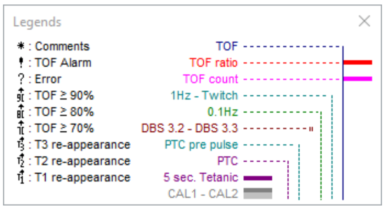

Legends and symbols can be shown by displaying the Legend window controlled by the legend icon 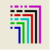 in the tool bar.

The special symbols above the graph in the Graphics Window are:

✱ Means that a comment has been entered by the user.

❢ Means that the TOF3D has detected a TOF alarm.

? Means that an error was reported.

↑90 First of three consecutive TOF results of [40 -100] % or higher (RP: three consecutive TOF results of less than or equal to 25%).

↑80 First of three consecutive TOF results of [35 - 95] % or higher (RP: three consecutive TOF results of less than or equal to 25%).

↑70 First of three consecutive TOF results of [30 - 90] % or higher (RP: three consecutive TOF results of less than or equal to 25%).

↑T3 First of three consecutive TOF results of 3 counts or higher (RP: three consecutive TOF results of less than 3 counts).

↑T2 First of three consecutive TOF results of 2 counts or higher (RP: three consecutive TOF results of less than 2 counts).

↑T1 First of three consecutive TOF results of 1 count or higher (RP: three consecutive TOF results of less than 1 count).

RP: Relaxation point must be present before a given recovery marker is shown.

The Graphics Window can also contain a temperature curve in the area between 100% twitch height and the top border line. The upper and lower temperature limits of the temperature curve correspond to the recorded temperature range.

Note:
If the TOF3D is still connected then after 30 seconds of inactivity the cursor will automatically jump to the last recorded measurement!!

Comments
Small comments can be placed simply by typing in letters in the marker box below the Graphics Window. Pressing any valid letter will initiate a comment for editing for the selected record. Pressing Enter or Esc while the marker box is focused will insert the text as a comment and return the focus to the Info Window. If a comment has been inserted, a * appears in the 2nd column and also above the related event in the Graphics Window.

Note:
Empty comments could mean that important clinical information was not filled in properly. Therefore the Save file / Save as dialog will display a red warning text if the file contains empty comments.

File tabs
Click on the file tabs to switch between open files. This can also be achieved on the Windows Menu.

Note:
The active recording file will be marked with brackets ('> filename <')

Status bar

The status bar can be toggled on and off by clicking on the tool bar and will show the status of the TOF3D in recording mode and facilitate remote control of the device.

For more information on the Status bar please see Options|Status bar.

Timer on/off
This button controls the visibility of the timer/alarm section of the toolbar. If pressed, the toolbar will expand in order to display the timer/alarm section.

Note:
In case an active guidance alarm sounds while the timer/alarm section is hidden the toolbar will automatically expand to show the guidance text.

Interval time
The current timer value is displayed here. The timer always starts from 00:00 and counts up every second. The maximum count is 99:59.

Timer start/stop
In stop-watch mode this button controls the start and stop of the timer.

In guided alarm mode, this button initializes the guided alarm sequence by starting the interval timer and by inserting measurement related comments for each sounding alarm. After activation of the guided alarm sequence, this button will become disabled (grayed) as long as the sequence is active and the only way to stop and reset the guided interval sequence, is by means of the Alarm on/off button.

Timer Reset
In stop-watch mode this button resets the timer to 00:00. The button will become disabled (grayed) if the guided alarm sequence is active.

Alarms on/off
Enables or clears the guided alarm sequence. The only way to stop a running active guide sequence is to turn the alarm off by activating this button.

Guided alarm text
Initially after the guided alarm has been turned on, the top line will show the first active guidance text in black and the bottom line will show the next guidance text in grey. Placing the mouse cursor over the box will produce a tool-tip indicating the complete guided alarm sequence (active alarm is marked by a *).

During the active guided sequence this box will contain both the current and the next guided alarm text. When an alarm sounds due to elapsed time interval the next active guidance text will be moved to the top and shown in red together with an audible signal. Then the next upcoming alarm text will appear in grey below. The end of a guided sequence will be indicated by the word "End".

Adjust initial alarm guide
Normally a guided alarm sequence will start with the first alarm in the programmed list, but by means of and the start point of the sequence can be set at any point in the list. This could be useful in case the operator accidentally forgot to start the guided alarm sequence at the appropriate time.

Note:
Adjustment of initial alarm guide is not possible during an active guided alarm sequence.

Check alarm guide
Every sounded alarm will be accompanied by a red guidance text. This text is meant as an instruction to the user. When the user has carried out the instruction he can check/accept the guided alarm by setting the check-mark next to the guidance text. This reverts the red guidance text back to black and will help him in remembering that he has carried out the instruction. The check-mark is automatically cleared when a new alarm is sounding.

Real time clock
If selected in the Options|Environment the program will display the time. Please note that changing the PC time will not influence the TOF3D device time stamps during an active recording session.

Edit Marker
By hitting a valid character the cursor will be frozen on the current event and the focus will change to the edit marker box. The marker edit box provides an easy way of making comments about a certain recorded event.. Each marker inserted will generate a * in both the Info Window as and in the Graphics Window. Press Enter or Esc to terminate editing, insert the comment and release the cursor.

Keyboard shortcuts for the File Window

Page Up	Scroll the Info Window up by one page and moves the cursor accordingly.
Page Down	Scroll the Info Window down by one page and moves the cursor accordingly.
↑	Scroll the Info Window up by one step and moves the cursor one step to the left.
↓	Scroll the Info Window down by one step and moves the cursor one step to the right.
Home	Select the first row of the file and moves the cursor to the beginning of the graph.
End	Select the last row of the file and moves the cursor to the end of the file.
Ctrl + Page Up	Select the top row in the visible part of the Info Window.
Ctrl + Page Down	Select the bottom row in the visible part of the Info Window.
Ctrl + ↑	Scroll the visible Info Window up without moving the cursor.
Ctrl + ↓	Scroll the visible Info Window down without moving the cursor.
Ctrl + Home	Select the first cell of the info window.
Ctrl + End	Select the last cell of the Info Window.
Shift + Home	Select the first cell of the selected row in the info window.
Shift + End	Select the last cell of the selected row in the Info Window.
Space	Open up the standard comments dialog box as an easy way of selecting between the 20 user defined standard comments.
Insert	Insert an empty comment (*) .
Enter	Alternative way of inserting an empty comment (*).
F1	Help.
F2	Create a new file.
F3	Open file.
F4	Save file.
Ctrl + F4	Close file.
F5	Export file information.
F6	Display file information.
F7	Search for the previous mark in the file. (user comment, recovery marker, TOF alarm, error)
Ctrl + F7	Search for the previous user comment or recovery marker in the file.
F8	Search for the next mark in the file. (user comment, recovery marker, TOF alarm, error)
Ctrl + F8	Search for the next user comment or recovery marker in the file.
F9	Search for the previous TOF alarm in the file.
F10	Search for the next TOF alarm the file.
F11	Search for the previous error in the file.
F12	Search for the next error in the file.
**Any letter**	Initiate marker editing - any valid letter or digit will appear in the marker box when typed on the keyboard.

Note:
If the TOF3D is still connected then after 30 seconds of inactivity the cursor will automatically jump to the last recorded measurement!!

File Menu

Command	Keyboard	Tool bar	Description
New file	F2		Create a new file and initiate recording mode.
Open file	F3		Display a file on the monitor, export a file to ASCII format or print out the file.
Save file	F4		Display the Save file menu from where you can save the current file.
Save as			Display the Save as menu from where you can save a file under a different name.
Close file	Ctrl + F4		Close the active File Window.
Export file	F5		Display the Export file menu from where you can export the current file to text format for further processing.
File info	F6		Display information about the file.
Print all			Print all available information. Both a graphic image as well all numerical data including an audit trail will be printed in one action. This function is also accessible through the Pop-Up menu.
Print info window			Print selected rows in the Info Window or print an Audit Trail. This function is also accessible through the Pop-Up menu.
Print graphic window			Print the visible section of the graph contained in the graphic windows. The print out can be made at various resolutions. This function is also accessible through the Pop-Up menu.
Print Screen			Print the desktop (entire window area of the TOF3D Monitor).
Printer Setup			Windows standard Printer Setup dialog box.
Log out			Log out from the current session.
Exit	Alt + F4		Close all windows and exit the program.

File|New file

This dialog box is used to create a file to contain recorded data from the TOF3D Monitor.

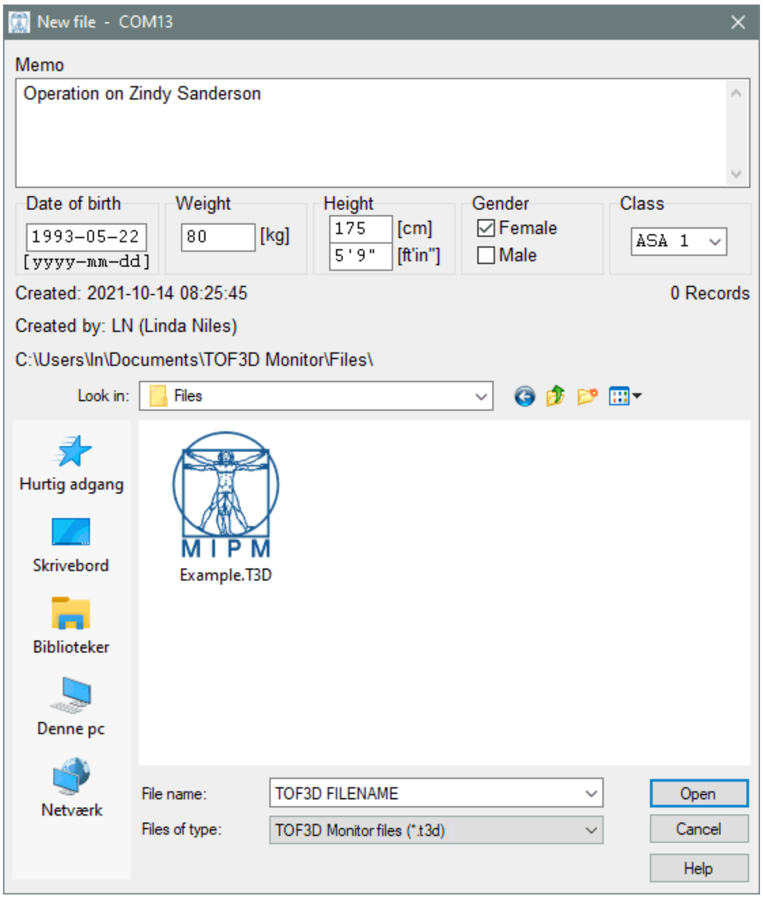

Look in
Select the directory designating the location of the file you want to use.

Note:
Supervisor can restrict available directories and in this case the dialog will display red warning text.

Directory actions
Use buttons to navigate/create directories and change display mode of current directory.

Files
This list box shows the files in the current directory. The program does not allow overwriting of existing files.

File name
Type in the name of the file you are about to record. You do not need to type the '.T3D' extension since this will automatically be added by the program. The program does not allow overwriting of existing files.

Files of type
This field determines which file types are to be included in the Files list box. Only extension *.T3D is allowed.

Memo
In the Memo field you have the option to enter some notes about the specific file. These notes will be saved along with the TOF3D data. The memo can be edited in recording mode, but also after the file has been saved.

Note:
An empty file memo or empty comments could mean that important clinical information was not filled in properly. Therefore the dialog will display a red warning text if the file contains an empty file memo or empty comments.

Date of birth
The subject's date of birth can be entered here. Please observe the date format below the entry box.

Note:
If the color of the entered text is red then the program will not accept and store the entered date.

Weight
The weight of the subject can be entered here with up to two decimal digits - max weight 300kg.

Height
The height of the subject can be entered here - max 255cm or 8'4".

Gender
The gender of the subject can be set here.

Class
ASA Physical status classification as defined by American Society of Anesthesiologists can be entered here.

Created
The creation time and date of the selected file.

Created by
The user ID (full name) of the user responsible for recording the selected file.

Records
The number of stored records/measurements in the selected file (not relevant for a new file).

Open button
By pressing this button, the program creates a file, with the name specified in the File Name field, and tries to initiate recording mode.

Cancel button
By pressing this button, the dialog box will be closed without creating a file.

Note:
Before recording can start, please set up the port to which the TOF3D is connected (Options|Environment).

File|Open File

This dialog box is used to load files for printing and post commenting.

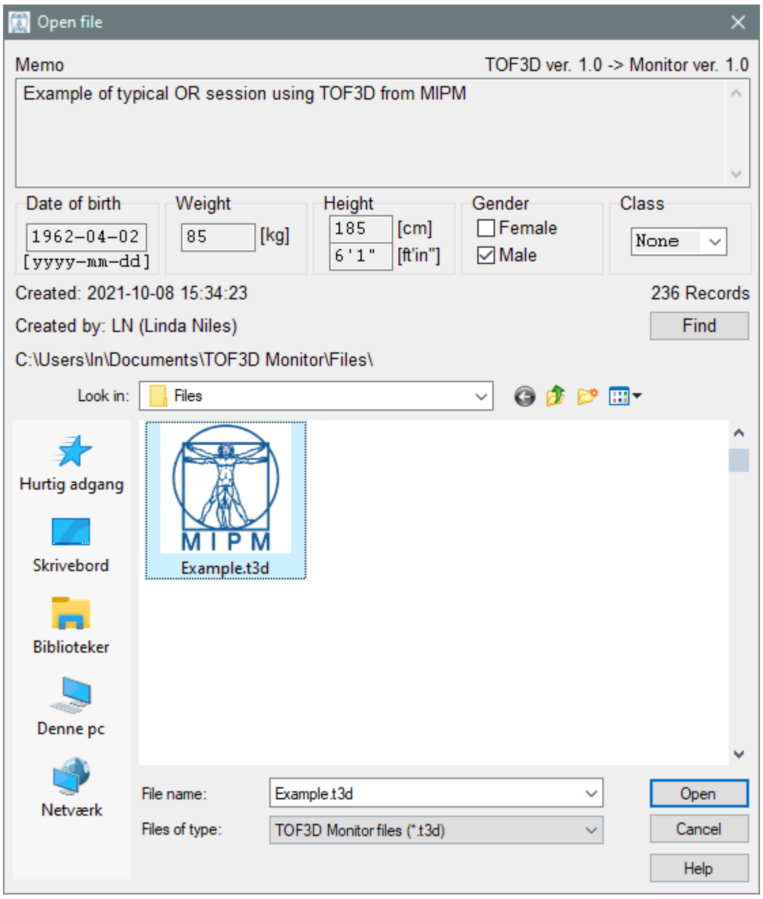

Look in
Select the directory designating the location of the file you want to use.

Note:
Supervisor can restrict available directories and in this case the dialog will display red warning text.

Directory actions
Use buttons to navigate/create directories and to change display mode of the current directory.

Files
This list box shows the files in the current directory. Furthermore, the contents of the selected file's Memo field and demographic data are shown above this file list. To search for a specific file name in the directory or its subdirectories please use the Find button.

File name
Shows the filename of the selected file

File of type
This field determines which file types are to be included in the Files list box. Only extension *.T3D is allowed.

Memo
The Memo field displays the notes about the currently selected file.

Versions
The versions of equipment originally used in the recording of the selected file.

Date of birth
The subject's date of birth.

Weight

The weight of the subject.

Height
The height of the subject.

Gender
The gender of the subject.

Class
ASA Physical status classification as defined by American Society of Anesthesiologists.

Created
The creation time and date of the selected file.

Created by
The user ID (full name) of the user responsible for recording the selected file.

Records
The number of stored records/measurements in the selected file.

Find button
Press this button to open the Find files dialog.

Open button
Press this button, and the selected file will be opened and displayed in the File Window.

Cancel button
By pressing this button, the dialog box will be closed without opening any files.

Find files dialog
This dialog makes it possible to search for files. The search criteria must be entered before pressing the Search button.

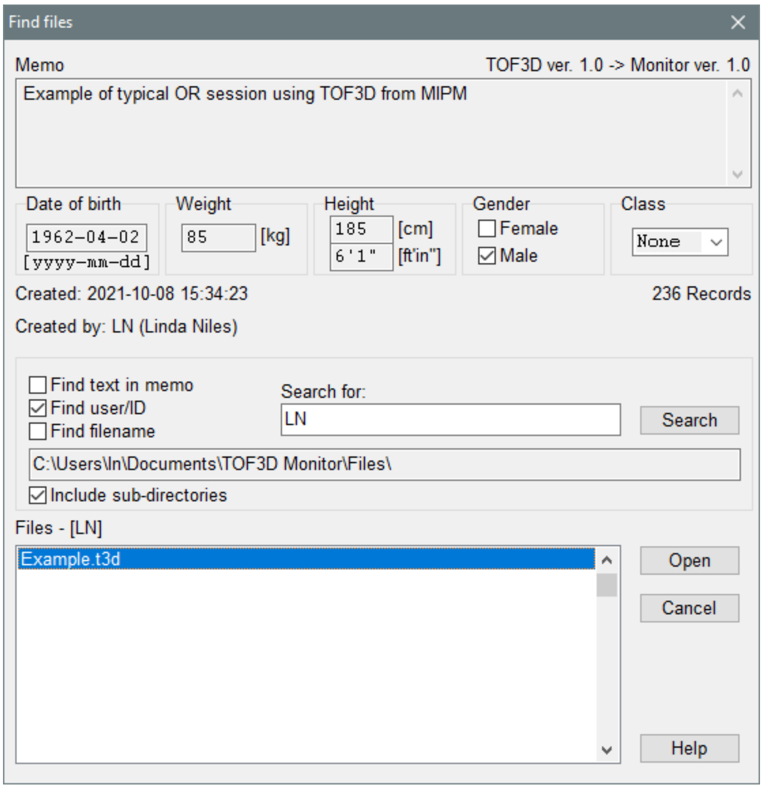

Search button
Start or stop the file search process. The accumulated search result will appear in the Search results box below.

Search text
Type in the text you want to search for, select which items to include in the search and click the Find button.

Include sub-directories
Check this if you want to include all underlying sub-directories in your search.

Find text in memo
Check this if you want to include a search for files with a specific phrase in their memo fields.

Find User/ID
Check this if you want to include a search for files recorded by a specific user.

Find filename
Check this if you want to include a search for a specific file name.

Search directory
The top level search directory is indicated here. This directory is the same as the current directory in the Open file dialog. Changing this directory is only possible in the Open file dialog.

Search results
Files that meet the search criteria will be listed here. Double clicking a file will open and display the file in the File Window.

Open button
Press this button, and the selected file will be opened and displayed in the File Window.

Cancel button
By pressing this button, the dialog box will be closed without opening any files and the Open file dialog will re-appear.

File|Save File

The window is used to edit memo and demographic data before saving a file.

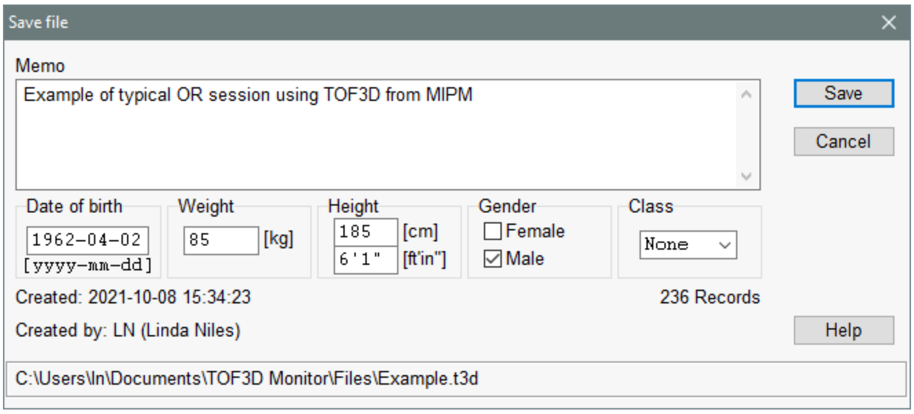

Memo
Here you can enter or edit notes about the file before the file is saved.

Note:
An empty file memo or empty comments could mean that important clinical information was not filled in properly. Therefore the 'Save file' dialog will display a red warning text if the file contains an empty file memo or empty comments.

Date of birth
The subject's date of birth can be entered here. Please observe the date format below the entry box.

Note:
If the color of the entered text is red then the program will not accept and store the entered date.

Weight
The weight of the subject can be entered here with up to two decimal digits - max weight 300kg.

Height
The height of the subject can be entered here - 255cm or 8'4".

Gender
The gender of the subject can be entered here.

Class
ASA Physical status classification as defined by American Society of Anesthesiologists can be entered here.

Created
The creation time and date of the file.

Created by
The user ID (full name) of the user responsible for recording the file.

Records
The number of stored records/measurements in the file.

File name
Name of the file to be saved.

Save button
By pressing this button, the dialog box will be closed and the file will be saved.

Cancel button
By pressing this button, the dialog box will be closed and the previous operation will be canceled.

No button
Warning by pressing this button you choose not to save changes you have made to the file.

Note:
This "No" option is not available if you are in recording mode.

File|Save as

This window is used to edit memo and demographic data before saving a file under a different name.

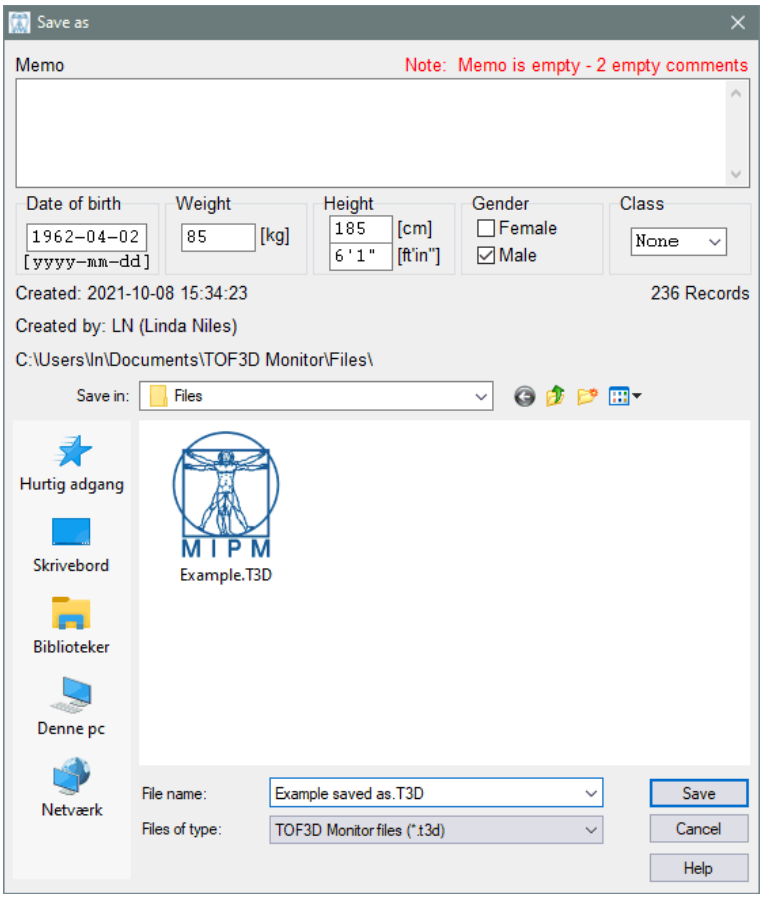

Memo
Here you can enter or edit notes about the file before the file is saved.

Note:
An empty file memo or empty comments could mean that important clinical information was not filled in properly. Therefore the dialog will display a red warning text if the file contains an empty file memo or empty comments.

Date of birth
The subject's date of birth can be entered here. Please observe the date format below the entry box.

Note:
If the color of the entered text is red then the program will not accept and store the entered date.

Weight
The weight of the subject can be entered here with up to two decimal digits - max weight 300kg.

Height
The height of the subject can be entered here - max 255cm or 8'4".

Gender
The gender of the subject can be entered here.

Class
ASA Physical status classification as defined by American Society of Anesthesiologists can be entered here.

Created
The creation time and date of the file.

Created by
The user ID (full name) of the user responsible for recording the file.

Records
The number of stored records/measurements in the file.

Save in
Select the directory designating the location of the file you want to use.

Note:
Supervisor can restrict available directories and in this case the dialog will display red warning text.

Directory actions
Use buttons to navigate/create directories and to change display mode of the current directory.

Files
This list box shows the files in the current directory.

File name
Here you can enter the new file name.

Note:
You are not allowed to overwrite existing T3D files.

Files of type
This field determines which file types are to be included in the Files list box. Only extension *.T3D is allowed.

Save button
By pressing this button, the dialog box will be closed and the file will be saved.

Cancel button
By pressing this button, the dialog box will be closed and the file will not change name.

File|Export File

The TOF3D Monitor saves all recorded data to an encrypted format under the file name specified by the user (with the extension 'T3D' automatically added). These data can be converted to a text file format (with the extension 'txt' automatically added). This can be done via the Export file item in the File menu.

To transfer your text file to a spreadsheet program, you either have to IMPORT your file or OPEN it, depending on the spreadsheet you use. You have to select the TAB separated format in your spreadsheet import menu ('TAB separated' means that fields are separated by the TAB character). If your spreadsheet has no option for 'TAB separated format' on its import menu, try 'number format' or other options to find out which import method works best with your spreadsheet.

For detailed information on importing files refer to the TOF3D Monitor Database Export Format and to the spreadsheet program manual.

This dialog box appears with the target directory for the export file and needs to be confirmed by pressing the OK button

The export location is defined in the Options|Environment, but can be temporary overruled by using this dialog box.

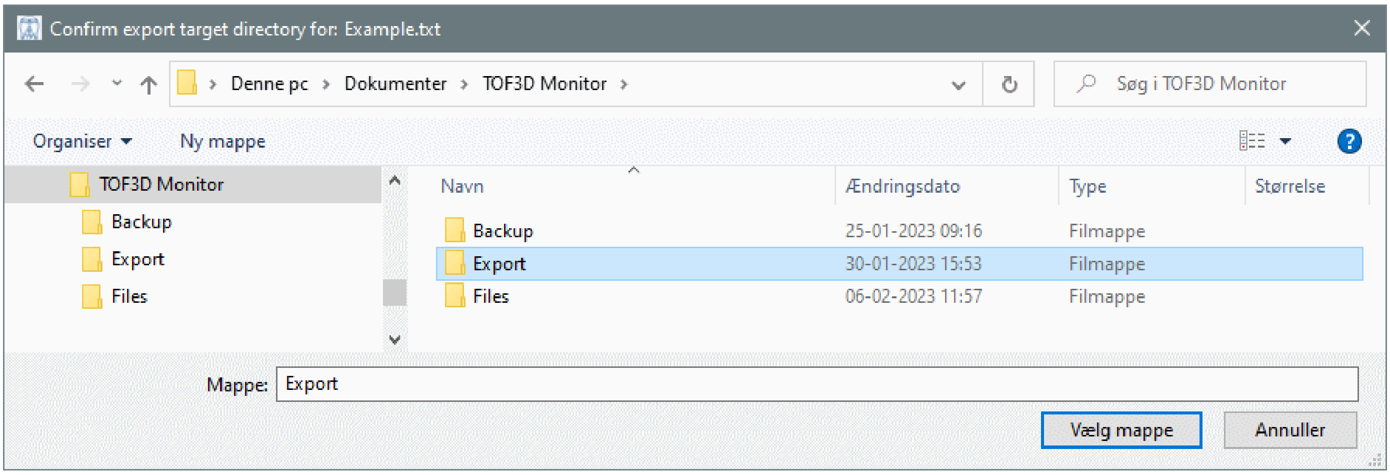

Confirm export target directory for:
The filename of the file to be created.

Directories
Select the directory designating the location of the export file to be created.

Make new folder
Click to create a new folder.

Select button
By pressing this button the export operation is performed.

Cancel button
By pressing this button the export operation is canceled.

File|File info

This window holds relevant information about the active file. The memo field and demographic data can be edited and saved.

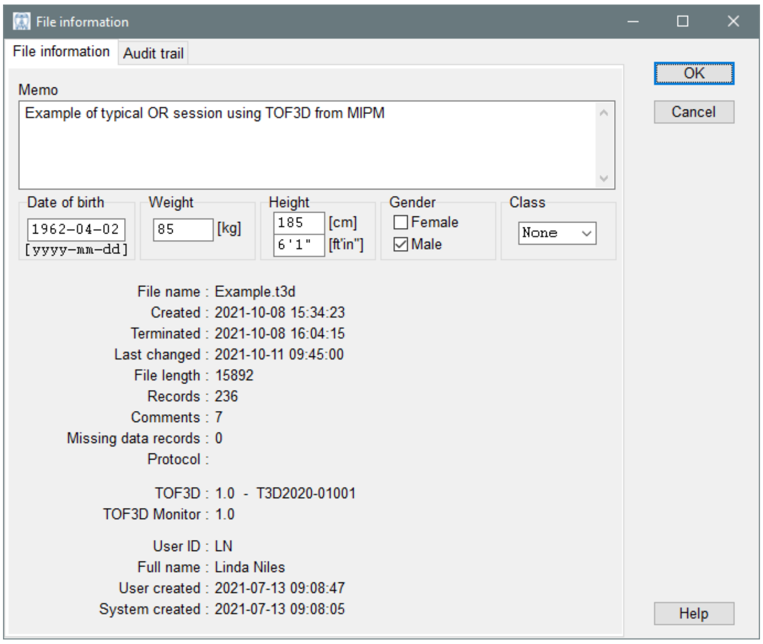

Memo
Contains the file memo and can if be modified if your user permissions allows for creation and saving of files.

Note:
An empty file memo or empty comments could mean that important clinical information was not filled in properly. Therefore the dialog will display a red warning text if the file contains an empty file memo or empty comments.

Date of birth
The subject's date of birth can be entered here. Please observe the date format below the entry box.

Note:
If the color of the entered text is red, then the program will not accept and store the entered date

Weight
The weight of the subject can be entered here with up to two decimal digits - max weight 300kg.

Height
The height of the subject can be entered here - max 255cm or 8'4".

Gender
The gender of the subject can be entered here.

Class
ASA Physical status classification as defined by American Society of Anesthesiologists can be entered here.

File name
The name of the current file. This name can be changed by saving the file under another name.

Created
The creation time and date of the file.

Terminated
The time of the last recorded measurement event.

Last changed
The time of the last modification of the T3D file.

File length
The total length of the file in bytes.

Records
The total number of records in the file.

Comments
The total number of user comments in the file.

Missing data records
The total number of missing data records. When connected to the TOF3D the Monitor will always try to download missing data records but if this process is prematurely terminated missing data records will reported here.

Protocol
The fixed protocol stamp stored in all files created by the system.

TOF3D
The software version of the TOF3D responsible for the data.

TOF3D Monitor
The software version of the TOF3D Monitor program responsible for receiving the original recording.

User ID
The user ID of the person responsible for the original recording.

Full name
The full user name of the person responsible for the original recording.

User created
The creation time of the person responsible for the original recording. This information is used to control the validation flags in the audit trail.

System created
The installation time of the TOF3D Monitor program responsible for the original recording. This information is used to control the validation flags in the audit trail.

OK button
Stores changes to the memo field and closes the window.

Cancel button
Discards all changes to the memo field and closes the window.

Audit trail

Select and view three possible audit trail formats:

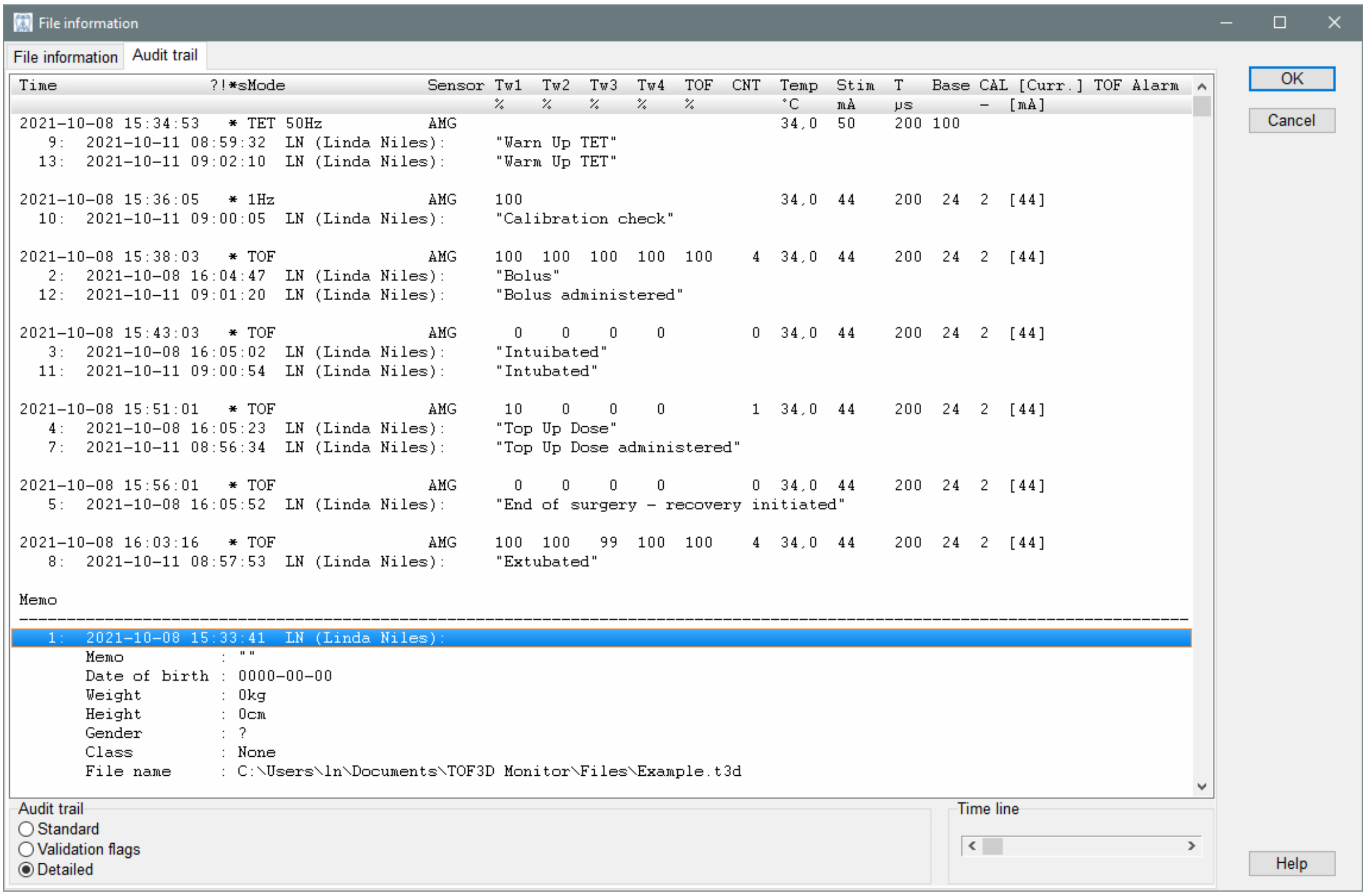

Standard

This Selection will result in a standard audit trail (with-out validation flags) of all actions done by the operator. This includes file name changes, demographic data, all comments and all corrections made to those. All changes will be marked with a time stamp.

The Audit trail can be used to document changes made during and after the actual recording of each event and could be very useful if someone by accident erased the text of an important comment.

Validation flags

This selection will cause validation flags to be printed on the audit trail. Validation flags are only printed in case one of two situations occurs:

[U] means that the user responsible for this comment looks identical to the user responsible for the original recording (same user ID), but has a different creation date.

[S] means that this comment is made on an installation different from the one responsible for the original recording.

Detailed

This selection will cause detailed validation flags to be printed on the audit trail. Validation flags are only printed in case one of two situations occurs:

[U 2020-01-12 10:26:49] means that the user responsible for this comment looks identical to the user responsible for the original recording, but was created at the time stated together with the validation flag. The creation time of the original user can be seen on the footer of the audit trail.

[S 2020-02-27 15:45:21] means that this comment is made on an installation created at the time stated together with the validation flag and different from the one responsible for the original recording. The creation time of the original installation can be seen on the footer of the audit trail.

Time line

Together with the time stamp each event in the audit trail is given a sequential number that will identify its order of appearance with-in the historic time line. This number makes it easier to follow the flow of events and changes in the audit trail.

Use the Time line scroll bar to step backwards and forwards in time in order to follow the order of appearance of each audit trail event. Sliding the Time line scroll bar all the way to the left, will set the blue cursor at the first recorded event in the audit trail. Sliding the scroll bar all the way to the right, will set the cursor at the last recorded event.

File|Print all

This dialog box controls the print-out of an entire file. The print-out contains all available graphical data, numerical data as well as an audit trail - if available.

The print-out is equipped with a time stamp and the output format will automatically be adjusted to the paper size.

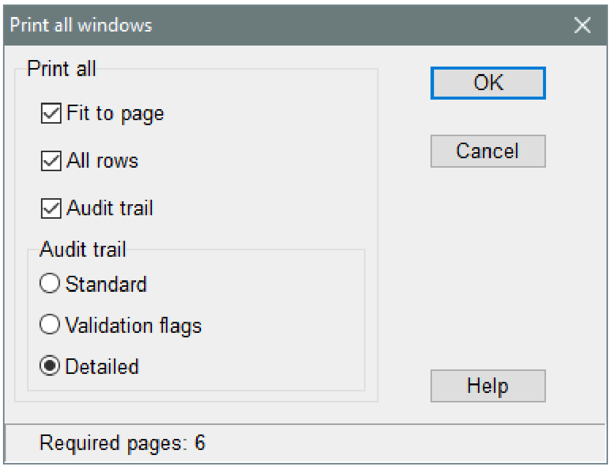

Print all
Shows which part of the file that will be included on the print-out.

Standard
This Selection will result in a standard audit trail (with-out validation flags) of all actions done by the operator. This includes file name changes, demographic data, all comments and all corrections made to those. All changes will be marked with a time stamp.

The Audit trail can be used to document changes made during and after the actual recording of each event and could be very useful if someone by accident erased the text of an important comment.

Validation flags
This selection will cause validation flags to be printed on the audit trail. Validation flags are only printed in case one of two situations occurs:

[U] means that the user responsible for this comment looks identical to the user responsible for the original recording, but has a different creation time.

[S] means that this comment is made on a TOF3D Monitor installation different from the one responsible for the original recording.

Detailed
This selection will cause detailed validation flags to be printed on the audit trail. Validation flags are only printed in case one of two situations occurs:

[U 2020-01-12 10:26:49] means that the user responsible for this comment looks identical to the user responsible for the original recording, but was created at the time indicated together with the validation flag. The creation time of the original user can be seen on the footer of the audit trail.

[S 2020-02-27 15:45:21] means that this comment is made on a TOF3D Monitor installation created at the time stated together with the validation flag and different from the one responsible for the original recording. The creation time of the original installation can be seen on the footer of the audit trail.

Status line
The status line reflects the required number of pages for the selected print-out. During the print-out the status line reflects the progress of the printing.

OK button
Starts printing.

Cancel button
Closes the dialog box or cancels a print-out.

File|Print info window

This dialog box controls the print-out of the info window. The print-out contains mode data, numerical data and user comments. The print-out is equipped with a time stamp and the output format will be adjusted to the paper size.

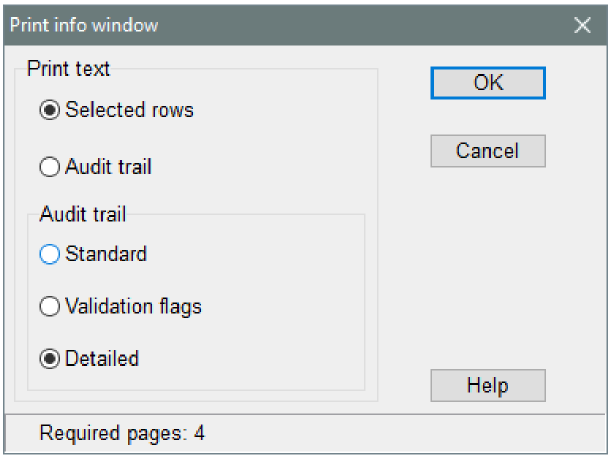

Print selected rows
Prints a copy of the selected rows in the info window.

The print-out will hold all visible data in the rows that have been selected by the user. To print the whole file first select all rows by clicking on 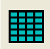 and then enter the Print info window dialog box by clicking on 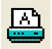 and click OK.

Print Audit trail
Prints an Audit trail of all entries made by the operators. This includes filename changes, demographic data, all comments and all corrections made to those. All changes are marked with a time stamp.

The Audit trail can be used to document all changes made during and after the actual recording of each event and could be very useful if someone by accident erased the text of an important comment.

Standard
This selection will cause a standard audit trail print with no validation flags.

Validation flags
This selection will cause validation flags to be printed on the audit trail. Validation flags are only printed in case one of two situations occurs:

[U] means that the user responsible for this comment looks identical to the user responsible for the original recording (same user ID), but has a different creation date.

[S] means that this comment is made on an installation different from the one responsible for the original recording.

Detailed
This selection will cause detailed validation flags to be printed on the audit trail. Validation flags are only printed in case one of two situations occurs:

[U 2020-01-12 10:26:49] means that the user responsible for this comment looks identical to the user responsible for the original recording, but was created at the time stated together with the validation flag. The creation time of the original user can be seen on the footer of the audit trail.

[S 2020-02-27 15:45:21] means that this comment is made on an installation created at the time stated together with the validation flag and different from the one responsible for the original recording. The creation time of the original installation can be seen on the footer of the audit trail.

Status line
The status line reflects the required number of pages for the selected print-out. During the print-out the status line reflects the progress of the printing.

OK button
Starts printing.

Cancel button
Closes the dialog box or cancels a print-out.

File|Print graphics window

This dialog box controls the print-out of the graphics window. The print-out will contain the currently visible graphical data. The print-out is equipped with a time stamp and the output format will automatically be adjusted to the paper size. You can select exactly what you want on your print-out by combining the scale and the actual window size before entering the Print graph window.

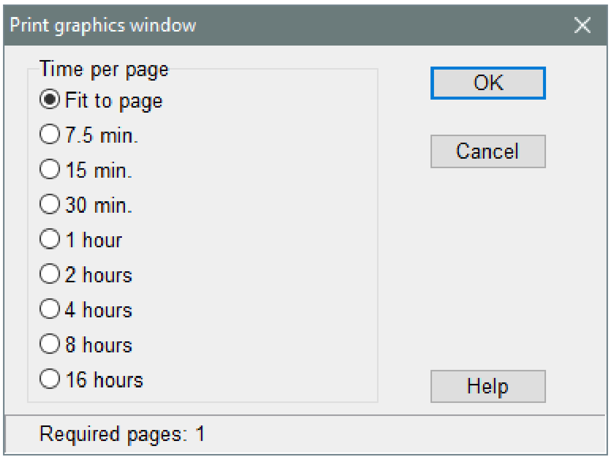

Fit to page
The currently visible part of the graphics window will be scaled so that it fits on one page.

Time per page
The currently visible part of the graphics window will be scaled so that one printed page equals the selected amount of time. Use these fixed time scales if you want to make comparable prints-outs, but watch out for the required number of pages presented in the status line.

Status line
The status line reflects the required number of pages for the selected print-out. During the print-out the status line reflects the progress of the printing.

OK button
Starts printing.

Cancel button
Closes the dialog box or cancels a print-out.

File|Log out

Logging out of the system will close all active file windows and bring up a new log in box:

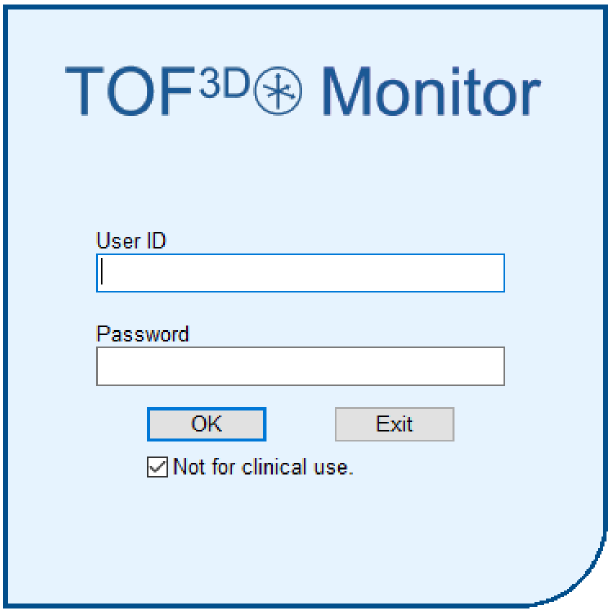

User ID
Type your user ID. Contact your Supervisor to get a user ID.

Password
Type your password. Contact your Supervisor if you have forgotten your password. The Supervisor will not be able to read or change your current password but he may completely delete your user account and re-create a new account with a default password for you. After having logged in for the first time it is wise to change your password in the Options|Users menu.

OK button
Press the OK button or Enter to log in to the system. You have 3 attempts before the program will be terminated. Please accept not to use the TOF3D Monitor program for clinical use.

Exit button
Press the exit button or Esc to exit the program.

Not for clinical use
Please tick to accept not to use this software as an active medical application.

Edit Menu

Command	Tool bar	Description
Copy Info Window		Copy the selected fields of the Info Window to the clipboard. The header fields are automatically added to the clipboard data. This function is also accessible through the Pop-Up menu.
Copy Zoom/Graphics Window		Copy the visible windows of the Zoom Window and the Graphics Window to the clipboard. This function is also accessible through the Pop-Up menu.
Toggle Fields		Choose between showing all possible fields in the Info Window and only showing the fields selected in the Options|File Window dialog box.
Select All		Select all fields in the Info Window for copying or printing purposes. This function is also accessible through the Pop-Up menu.
Reference Time		Toggles between absolute time and relative time to the current cursor location. Selection of relative time is indicated by brackets "[hh:mm:ss]" around the cursor time in the Graphics Window.
Insert comment		Insert a blank comment at current cursor position and prepare for editing. Alternatively use Insert or Enter to add a blank comment. You can edit the comment field in the normal way.
Zoom In		Zoom in on the current cursor position in the Graphics Window. The limit of magnification is a time resolution of four pixels per second (like the Zoom Window).
Zoom Out		Zoom out on the current cursor position in the Graphics Window. The limit is so that all data fits well inside the Graphics Window.
Zoom to selection		The graph will be scaled in such a way that at least all data of the selected cells will be visible in the Graphics Window. This function is also accessible through the Pop-Up menu.

Search Menu

Command	Keyboard	Tool bar	Description
Previous mark	F7	←	Search for the previous mark in the file<br>(user comment, recovery marker, TOF alarm, error)
Next mark	F8	→	Search for the next mark in the file.<br>(user comment, recovery marker, TOF alarm, error)
Previous comment	Ctrl + F7	Ctrl + ←	Search for the previous user comment or recovery marker in the file.
Next comment	Ctrl + F8	Ctrl + →	Search for the next user comment or recovery marker in the file.
Previous TOF alarm	F9		Search for the previous TOF alarm the file.
Next TOF alarm	F10		Search for the next TOF alarm in the file.
Previous error	F11		Search for the previous error in the file.
Next error	F12		Search for the next error in the file.

Options Menu

Command	Tool bar	Description
Environment		Dialog box to select the serial port of the personal computer to which the TOF3D is connected. "Auto save" and "Tool bar tips" etc. can also be controlled here.
File window		Dialog box to set up the way a file window is displayed, including visible Info, Zoom or Graphics Window, visible fields in the Info Window, visible temperature curve etc.
Legend window		Toggle the Legend Window on/off. This window contains legends of all twitch types that can appear in the Zoom and Graphics Window. The legend window is always on top of the File Windows and can be dragged to any position on the screen.
Status bar		Toggle the TOF3D status bar and the remote control panel on and off. Data will only appear when connection to the TOF3D has been established.
Standard Comments		Dialog box where 20 different and often used comments can be defined and stored. These user defined comments can later be inserted by pressing Space or clicking 0-19.
Timer		Toggle timer section of toolbar on and off.
Start timer		Start the timer or the guided alarm sequence
Reset timer		Reset the timer to 00:00
Alarms		Enable/disable guided alarms
Alarm guides		Open the alarm guides dialog box where a guided alarm sequence can be programmed.
Users		Dialog box where various user information can be edited. If you are logged in as Supervisor this option enables the creation and deletion of users.

Options|Environment

You use this dialog box to select the serial port to use for connection to the TOF3D. Also the Auto save function, Tool bar tips and various directory locations can be controlled here.

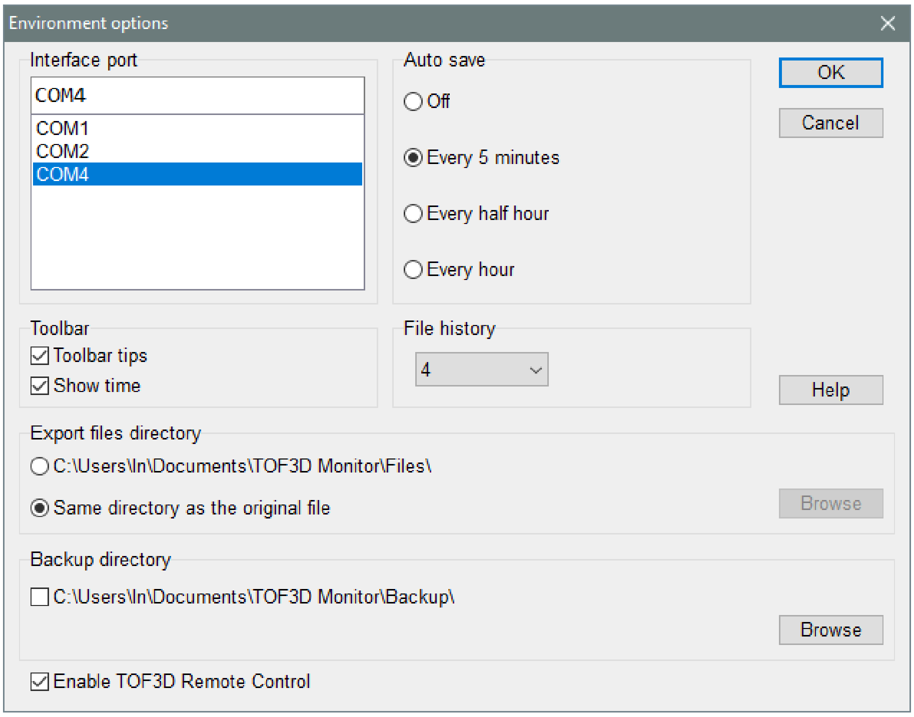

You must setup the program to use a dedicated COM port representing an installed a TOF3D-LINK interface also connected to the TOF3D device.

Interface port
This list contains all installed TOF3D-LINK interface ports and the header in combination with the blue bar indicates which port the program will use for connection to the TOF3D.

Click to select the port through which the TOF3D will be connected.

IMPORTANT:

When inserting the TOF3D-LINK interface into an USB port Windows will automatically assign a serial port name to the device. This port assignment can be seen in the above Interface port list and in Windows Device Manager:

Windows Control Panel / System / Hardware / Device Manager / Ports (COM & LPT).

To change the port number in the Device Manager click on the Serial USB Port (COMx) / Port Settings / Advanced and change the COM Port Number.

To prevent Windows from detecting the TOF3D as a serial mouse disable Windows "Serial Enumerator" under Serial USB Port (COMx) / Port Settings / Advanced / Miscellaneous Options. If Windows has falsely detected the TOF3D as a serial mouse this can be seen as random mouse cursor jumps.

Auto save

If not set to *Off* the Auto save function ensures that the all recorded data is automatically saved to disk at the given fixed intervals determined by the setting. This minimizes the risk of loosing valuable data during recording mode.

Note:
If a Backup directory has been enabled the auto save function will also auto save to the selected backup folder.

Tool bar tips
Toggle the tool bar tips (the help text bubble that appears below the icon approximately one second after the mouse cursor is placed on the button).

Show time
Toggle the display of the real-time on and off. The real-time clock will be displayed in the tool bar and will be scaled to fit the available free space in the tool bar.

File history
Sets the number of recently opened files to show in the file menu. The number can be set between 0 (off) and 10.

Export Files Directory
In this box you can choose where to save the export files. You can make the default destination either a fixed user defined export directory or the same directory as the original file.

If you select the user defined directory you have to specify the path in Export directory field, by using the related Browse button).

Backup Directory
In this box you can choose whether or not to double save files in a separate backup directory. If you select to use a backup directory you have to specify the path in Backup directory field, by using the related Browse button. Backup files are stored with full file path reflection so it is best not to use a deep path as Backup Directory.

Note:
Changing the backup directory settings can be disabled by the Supervisor.

Browse button
Press this button to open a browse dialog that lets you select either the default export directory or the backup directory.

Note:
Changing the backup directory settings can be disabled by the Supervisor.

Enable TOF3D Remote Control
Toggle the Remote Control panel on and off. The Remote Control panel is only active when connection to the TOF3D has been established.

OK button
Press this button to accept the settings in the dialog box, save them permanently (i.e. use them as the new standard options) and close the dialog box.

Cancel button
By pressing this button, the dialog box will be closed without making any changes.

Browse dialog

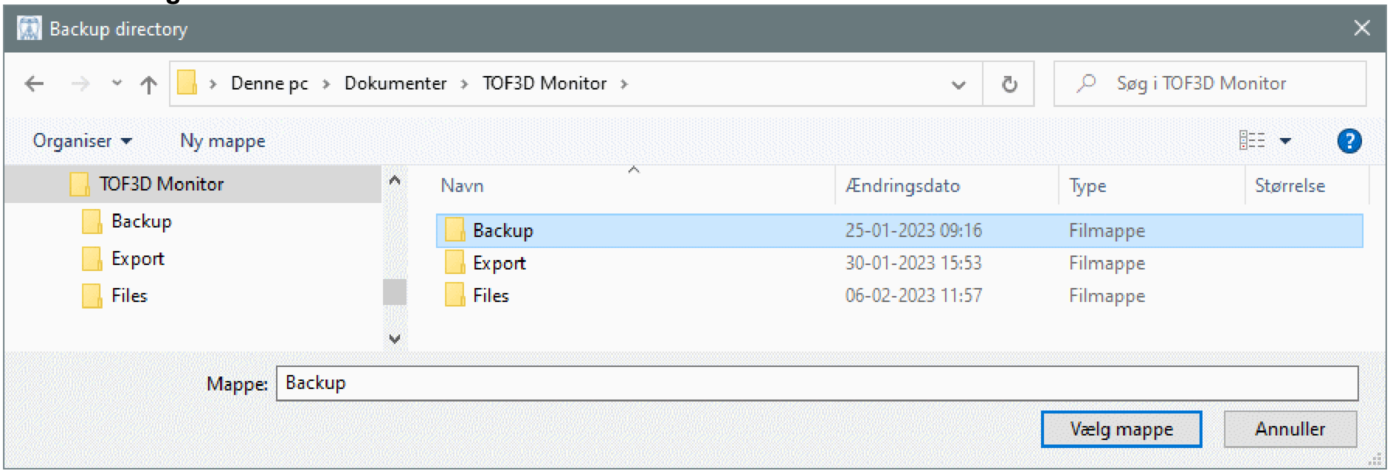

Directories
Select the destination directory for the default export directory or the backup directory

Make new folder
Press this button to create a new sub-directory in the currently selected directory.

Select button
Press this button to accept the selected export or backup directory and close the browse dialog box.

Cancel button
By pressing this button, the browse box will close without making any changes.

Options|File Window

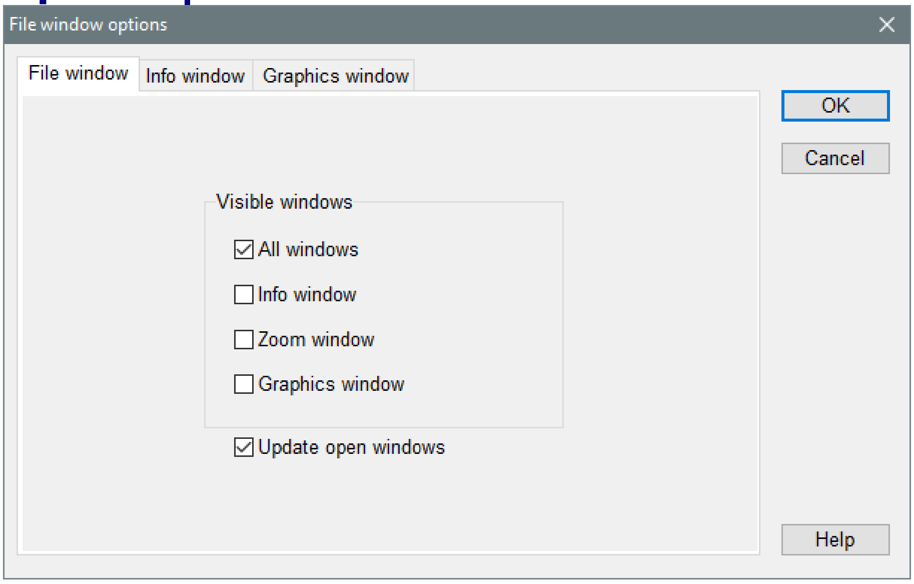

This dialog box is used to set up the way a File Window is displayed. The first page is a general set up of the File Window, the rest are for the windows within the File Window.

IMPORTANT:
The changes in the File Window setup will affect all open windows on the desktop unless you un-check Update open windows before clicking OK!

Visible Windows
Choose which windows should by default be visible in the File Window.

Update open windows
Un-check this box to prevent changing existing file windows. This box is automatically checked if you make changes to the visible windows setting.

OK button
Press this button to accept the settings in the dialog box, save them permanently (i.e. use them as the new standard options) and close the dialog box. Furthermore, all open File Windows will be updated according to the settings in this dialog box.

Cancel button
By pressing this button, the dialog box will be closed without making any changes.

Options|File window|Info window

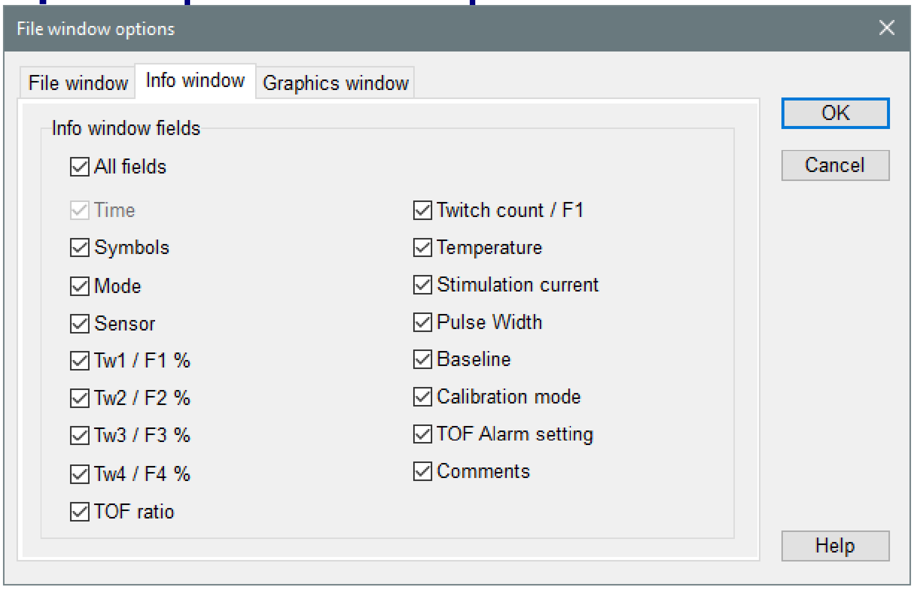

Choose which fields are to be visible in the Info Window.

Info Window Fields

All fields		All fields are shown in the info window
Time		The actual time of the recorded event. This field cannot be unchecked.
Symbols	*	Means that a user comment is present for the related event.
	?	Means that an error has been reported.
	!	Means that a TOF alarm has been reported.
Mode		Shows the stimulation type or mode.
Sensor		Shows attached sensor type.
Tw1 / F1 %		Twitch height in single twitch stimulation, and twitch height of first twitch in TOF modes.<br>In Tetanic MMG mode this column is used to show F1 (Fmax = 100%).
Tw2 / F1 %		Twitch height of second twitch in TOF modes.<br>In Tetanic MMG mode this column is used to show F2.
Tw3 / F1 %		Twitch height of third twitch in TOF modes.<br>In Tetanic MMG mode this column is used to show F3.
Tw4 / F1 %		Twitch height of fourth twitch in TOF modes.<br>In Tetanic MMG mode this column is used to show F4 (Fend).
TOF ratio		If four twitches are detected and the first twitch is at least 20%, the TOF ratio is calculated as: TOF ratio = (Twitch 4 / Twitch 1) x 100 [%].
Twitch count / F1		Shows the number of detected twitches (>= 3%) in a TOF stimulation or the number of detected consecutive twitches (>= 3%) in a PTC stimulation.<br>In Tetanic MMG mode this column is used to show the absolute size of F1 (Fmax).
Temperature		The surface temperature measured in °C by the TOF3D can be displayed.
Stimulation current		Shows the stimulation current (0-60 mA).
Pulse width		Shows whether 300 µs or the normal 200 µs stimulation pulse width has been selected.
Baseline		The sensor baseline setting in the range [1 - 581].
Calibration Mode		Shows the current calibration status together with the calibration current. In CAL mode 1 the current states the current used in the calibration procedure. For CAL mode 2 the supra maximal current found by the TOF3D is shown. Non calibrated measurements will result in a blank field
TOF Alarm Setting		Show the TOF alarm setting (values outside the alarm window will generate a TOF alarm).
Comments		Show user editable comments related to a recorded event.

OK button
Press this button to accept the settings in the dialog box, save them permanently (i.e. use them as the new standard options) and close the dialog box. Furthermore, all open file windows will be updated according to the settings in this dialog box.

Cancel button
By pressing this button, the dialog box will be closed without making any changes.

Options|File Window|Graphics Window

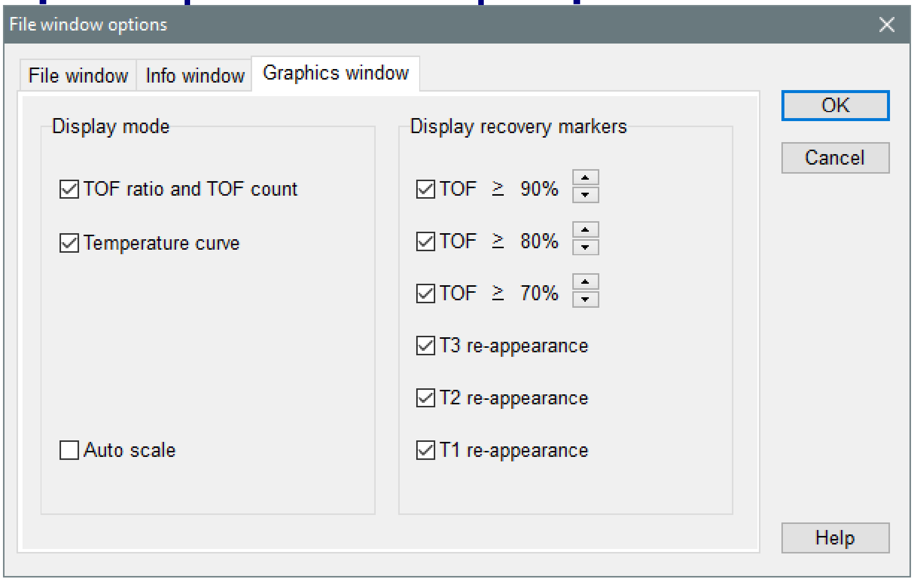

TOF Display Mode
Controls if data are presented in either Normal Mode or Trend Mode (showing TOF ratio and TOF count).

Temperature Curve
If checked and temperature data is available, the temperature curve is plotted in the Graphics Window.

Auto scale

If checked, the Auto scale function scales the entire graph so that the complete graph can be seen in the Graphics window. The Auto scale function is only active if the cursor is positioned at the last recorded event. This is to allow the user to do a manual zoom on the graph while in recording mode.

Note:
Press the END button to place the cursor at the last recorded event in order to re-activate auto scaling.

Recovery markers
If checked, the relaxation curve plotted in the Graphics Window will be equipped with recovery markers. By means of the up/down buttons, the TOF ratio recovery markers can be adjusted between 30% and 100% in steps of 5%. The top marker will always be the marker closest to a TOF ratio of 100% and if one TOF ratio marker is about to be adjusted below or above another TOF ratio marker, this other marker will be unchecked and pushed up/down accordingly.

A recovery marker is only plotted if a relaxation point (RP), given as required relaxation degree, has been detected prior to a given recovery point.

These markers are:

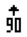 First of three consecutive TOF results of [40 -100] % or higher
(RP: three consecutive TOF results of less than or equal to 25%).

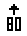 First of three consecutive TOF results of [35 - 95] % or higher
(RP: three consecutive TOF results of less than or equal to 25%).

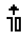 First of three consecutive TOF results of [30 - 90] % or higher
(RP: three consecutive TOF results of less than or equal to 25%).

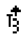 First of three consecutive TOF results of 3 counts or higher
(RP: three consecutive TOF results of less than 3 counts).

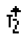 First of three consecutive TOF results of 2 counts or higher
(RP: three consecutive TOF results of less than 2 counts).

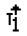 First of three consecutive TOF results of 1 count or higher
(RP: three consecutive TOF results of less than 1 count).

By default the program is set to detect recovery for T2, T3 and TOF ratio 70%, 80% and 90%.

Note:
Changing the recovery marker settings can be disabled by the Supervisor.

OK button
Press this button to accept the settings in the dialog box, save them permanently (i.e. use them as the new standard options) and close the dialog box. Furthermore, all open File Windows will be updated according to the settings in this dialog box.

Cancel button
By pressing this button, the dialog box will be closed without making any changes.

Options|Legend Window

The Legend Window is a legend of line styles and colors used for different stimulation types in the graphs.

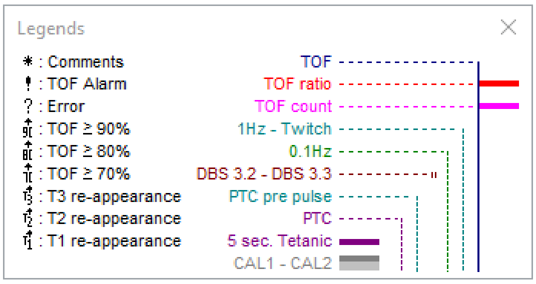

The legend window can be toggled on and of by clicking on 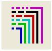 in the tool bar.

Every type of stimulation is represented in the graphics window by colored vertical lines.

A red TOF ratio bar represents the calculated TOF ratio - T4/T1 [%].

The number of present pink TOF count bars represent the number of detected TOF twitches in case the TOF ratio cannot be calculated.

The elevation of a purple bar depicts the stimulation strength [mA] of a 5 sec. Tetanic stimulation used in TET & PTC.

Where no patient response has been recorded elevated small vertical dots depicts the stimulation strength [mA] for the given stimulation.

The special symbols in the left column are:

*	Means that a comment has been entered by the user.
!	Means that a TOF alarm has been reported.
?	Means that an error has been reported.
90	First of three consecutive TOF results of [40 -100] % or higher<br>(RP: three consecutive TOF results of less than or equal to 25%).
80	First of three consecutive TOF results of [35 - 95] % or higher<br>(RP: three consecutive TOF results of less than or equal to 25%).
70	First of three consecutive TOF results of [30 - 90] % or higher<br>(RP: three consecutive TOF results of less than or equal to 25%).
T3	First of three consecutive TOF results of 3 counts or higher<br>(RP: three consecutive TOF results of less than 3 counts).
T2	First of three consecutive TOF results of 2 counts or higher<br>(RP: three consecutive TOF results of less than 2 counts).
T1	First of three consecutive TOF results of 1count or higher<br>(RP: three consecutive TOF results of less than 1 count).
RP:	Relaxation point must be present before a given recovery marker is shown.

Options|Status Bar

The Status bar shows the status of the TOF3D in recording mode and if enabled in Options|Environment also allows for remote control of the TOF3D device.

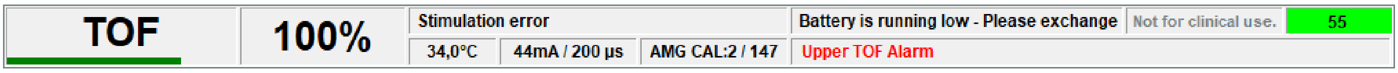

Except for the Status line and the Data Record status, data will only be shown if the Status bar is selected in Options|Status bar and when connection to the TOF3D has been established.

The status bar can be toggled on and off by clicking 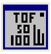 on the tool bar.

Operational mode
Operational mode, settings and stimulation countdown progress are shown here. The bottom bar acts as stimulation indicator and as a progress indicator for next stimulation.

Measurement result
Valid measurement results shown in % or in counts. When no objective measurement is at hand, this field is blank.

Stimulation Errror result
Various stimulation / calibration related errors reported by the TOF3D device will be shown here.

Status Line
This line is mainly used to provide error information reported by the TOF3D or by the monitor program. This line is always visible, also in the case where the status bar has been turned off.

Skin temperature
If available, the skin temperature will be shown here in °C, otherwise this field is blank.

Stimulation strength / pulse width
Dependent of the TOF3D display setting, the stimulation strength is shown in mA or µC and the pulse width is shown in µs.

Sensor Type / Calibration Mode / Sensor baseline
Sensor type is shown followed by calibration mode:

'CAL-' means non calibrated

'CAL1' means normal calibration

'CAL2' means supra maximal calibration

The sensor baseline is shown after the calibration mode and can be between 1 and 581.

TOF Alarm
When a TOF Alarm is reported by the TOF3D, this alarm will be indicated in red together with an audible sound.

Note:
You must enable the windows system sound for WAV files in Windows Control Panel in order to hear this alarm. You can test if audible alarms are working in the Options|Alarm guides menu.

Data Record status
The TOF3D stores all recordings internally in a non volatile memory. When connection has been established the TOF3D Monitor will automatically retrieve all stored data from the TOF3D.

The colored Data Record status can be used to follow this synchronization process:

RED:	48	The number indicates a large number data records NOT yet synchronized to the TOF3D Monitor.
YELLOW	14	The number indicates a small number of data records NOT yet synchronized to the TOF3D Monitor.
GREEN	55	The number indicates the total number of recorded and synced data records. All data records are synchronized.

Remote Control panels

If enabled in Options|Environment allows for setup and remote control of a connected TOF3D device.

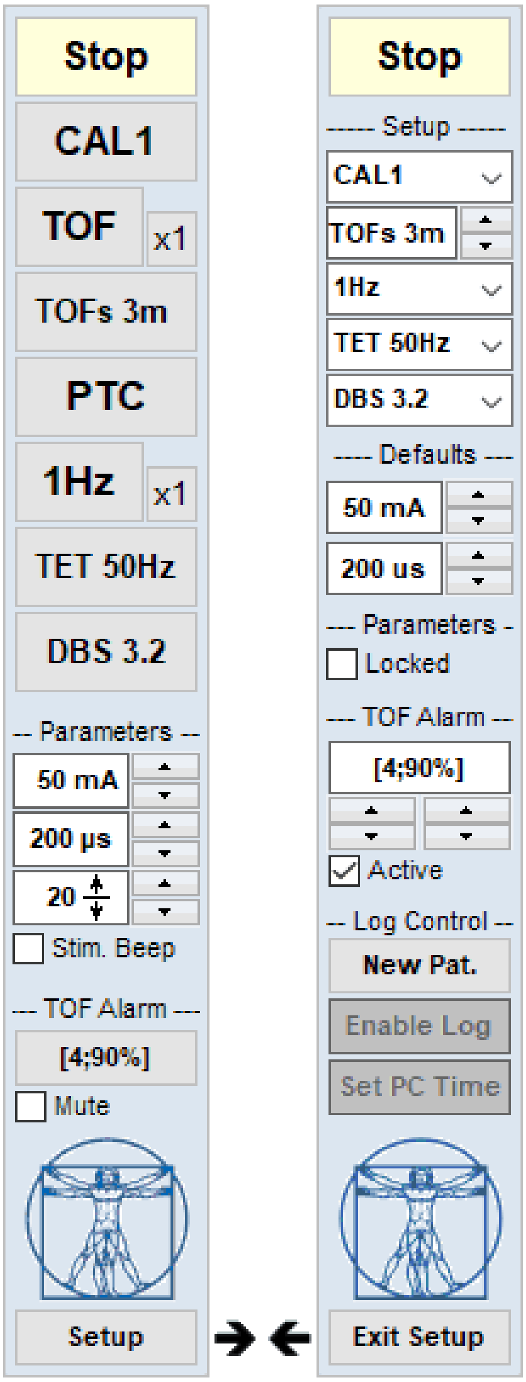

The Remote Control panel can be used to remote control a connected TOF3D device.

A MAIN control panel and a SETUP panel exists. The two panels mimics relevant parts of the TOF3D LCD User Interface.

MAIN Panel

- Click the *large buttons* to stop or the start continuous or single (x1) stimulations on the TOF3D device.
  The active mode will be indicated on the relevant button by a light yellow color.
- Click *Up/Down controls* to adjust relevant stimulation / measurement parameters:
  Stimulation current [mA], Pulse width [µs], Sensor Baseline.
- Click *Tick boxes* to turn On/Off relevant options:
  Stimulation Beep, TOF Alarm Muting.
- Click *Setup* to enter the Setup Panel.

Setup Panel

- Click the *Drop down boxes* to setup the available stimulation modes of the TOF3D device.
  Note: Some stimulations can be set to *Off* and will then NOT appear on the MAIN Panel nor be selectable on the TOF3D device.
- Click *Up/Down controls* to adjust relevant default parameters or to adjust the TOF Alarm levels:
  Default stimulation current [mA], Default Pulse width [µs], TOF Alarm levels.
- Click *Tick boxes* to turn On/Off relevant options:
  Lock Parameters, TOF Alarm On/Off.
  Note: Parameters can be *Locked* and will then NOT be adjustable on the MAIN Panel nor on the TOF3D device.
- Click *New Pat.* to erase data and prepare TOF3D device for a new patient. A confirmation dialog appears before this operation is carried out.
- Click *Enable Log* to allow logging data to the PC - Only possible if Log is disabled in the TOF3D device.
- Click Set PC Time to synchronize the PC clock to the TOF3D device. This is only possible when no data has been recorded and the TOF3D device is in Stop mode
- Click *Exit Setup* to revert back to the MAIN Panel
  Note: After 30 seconds of inactivity the SETUP panel is automatically exited.

Options|Standard Comments

As a help in the daily work a set of 20 pre-defined comments can be accessed by pressing Space or by means of the tool bar icon.

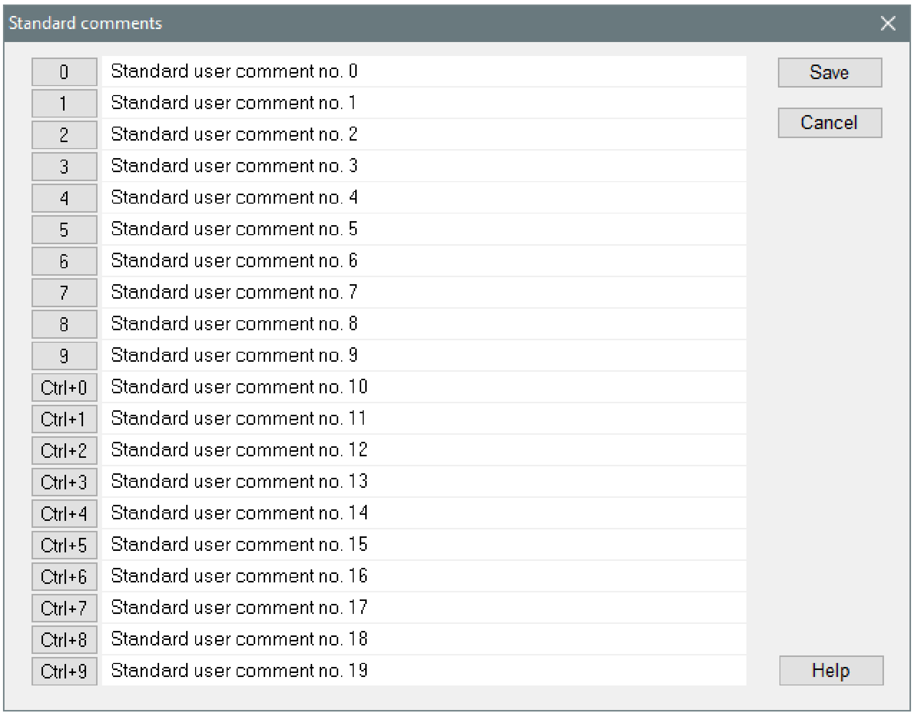

Speed Keys
Press one of the keys (mouse or 0 - 9) to insert a pre-defined comment into your file. Pressing Ctrl + 0 - 9 will select Standard user comment no. 10 - 19. The window will close when a standard comment is inserted.

Note:

If the dialog has been opened by means of Space or 0-19 then comments can still be inserted by means of Insert or Shift + Enter.

Standard comments
Edit the pre-defined comments.

WARNING:
All users will have access to the same set of pre-defined comments.

Save button
Pressing this button will make any changes to the standard comments permanent.

Cancel button
By pressing this button, the dialog box will be closed without making any changes to the standard comments.

Options|Alarm guides

In order to help investigators or study staff to remember various routine jobs during a recording session, this menu enables the user to program up to 20 alarm intervals each with an associated guidance text.

When the user starts a guided alarm sequence, the first active guidance text is inserted as a normal comment in the recording file and the timer is re-started from 00:00. When the pre-programmed interval time has elapsed, the next active alarm will sound and the associated guidance text will be shown and inserted as a new normal comment. The guidance text will be shown in red on the main toolbar. It is now possible to accept the guided alarm by clicking the check-mark to the right of the guidance text. This function is only meant as a help for the user and does not influence the sequence of guided alarms in any way. This sequence will continue until the last active guidance alarm has sounded.

Note:

Guided alarm comments will be inserted on the last know data record and not on any previous older records. Guided alarm comments will never overwrite existing user comments nor earlier guided alarms comments.

The intention is only to use guided alarms while logging data in real-time. If however the TOF3D Monitor is in the progress of downloading older records from the TOF3D device then Guided alarm comments will only appear in the T3D file in case the most recent record has been downloaded and contains no comment.

Warning:

Guided alarms should NOT be used for any patient critical tasks and are only intended to be used to guide investigators during clinical investigations or alike.

The dialog box below can be used to set up a complete guided sequence.

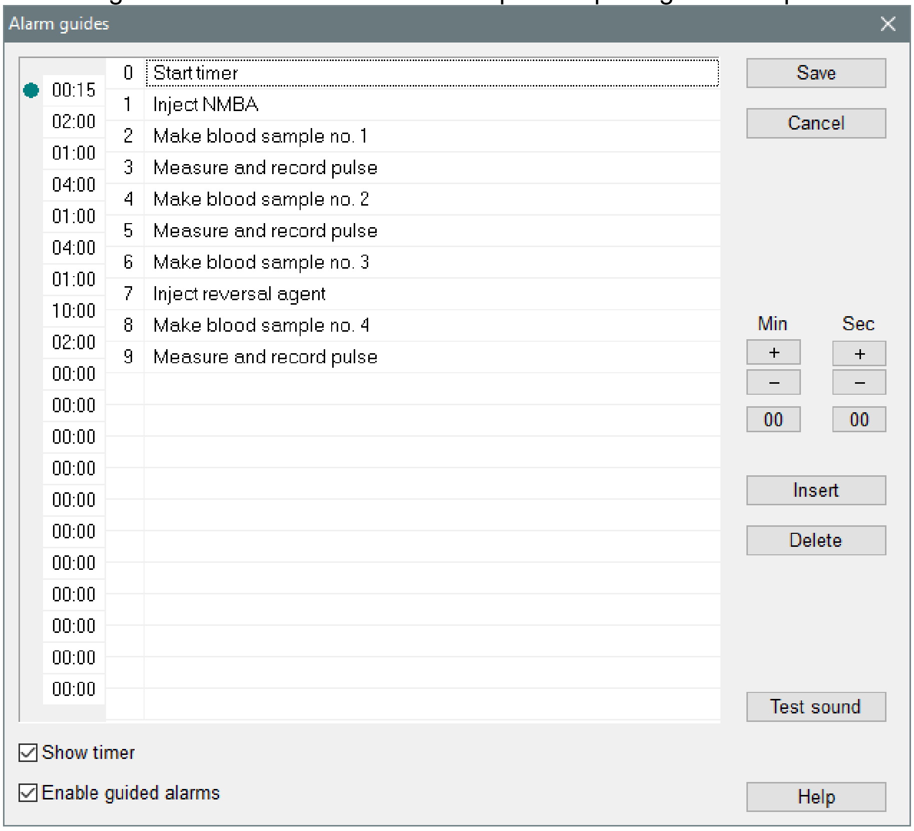

Guides

This contains user configurable guidance texts. If the guidance text is active then its ID number is shown to the left of the actual text. If no number is shown the guidance text is inactive. Guidance texts are only active if it is either the last one or has a related interval timing different from 00:00. The related interval time is marked with a green dot.

Interval time

The interval time can be adjusted between 00:00 (inactive) and up to 99 minutes and 59 seconds (99:59). The setting of the interval time is controlled on the right panel of the dialog.

Show Timer

This setting controls the default setting of the timer. If checked the main toolbar will extend to allow the timer to be visible.

Enable guided alarms
This setting controls the default setting of the guided alarm. If checked the guided alarm sequence will be enabled when the timer is visible.

Minutes up/down
Use the up/down to control the minutes of the interval time of the selected guidance text. Keeping the mouse down for a longer period of time will accelerate the advancement of the minutes. The value will wrap around if adjusted outside the limits [0; 99]

Minutes reset
This button will reset the minutes of the interval time of the selected guidance text to zero.

Seconds up/down
Use the up/down to control the seconds of the interval time of the selected guidance text. Keeping the mouse down for a longer period of time will accelerate the advancement of the seconds. The value will wrap around and increase/decrease the minutes, if adjusted outside the limits [0; 59]

Seconds reset
This button will reset the seconds of the interval time of the selected guidance text to zero.

Save button
Press this button to accept and save the programmed guided alarm sequence and close the dialog box.

Cancel button
By pressing this button, the dialog box will be closed without making any changes to the guided alarm sequence.

Insert button
This button will move the selected guidance text down and insert an empty row. Note that guides located on the bottom line will be deleted in order to make room for the inserted row.

Delete button
This button will delete both the selected guidance text and its related interval time.

Test button
Use this button to test if the audible alarm annunciation is at a proper audible level. If not please adjust windows general audio level and ensure that windows can play WAV files.

Options|Users

This option has two functions depending on which user accessing the menu.

The Supervisor can add, delete and changes permissions for other users.
Any NORMAL USER including the Supervisor can only edit personal information stored under his own user ID.

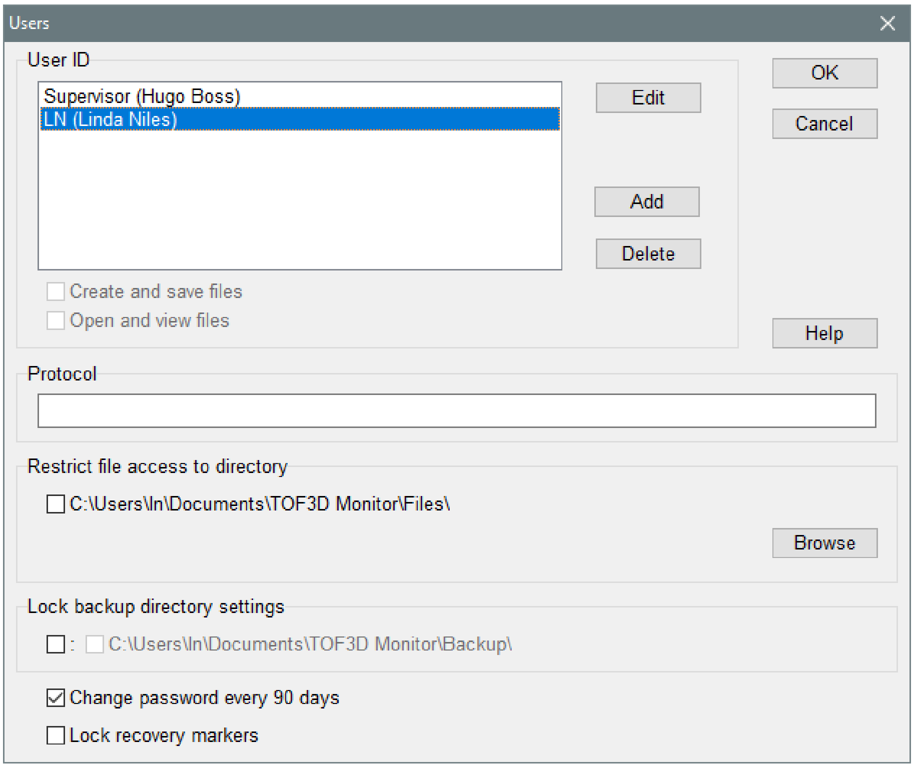

User ID
This is a list of all registered users in the system. Click Edit or Double click on your own name to open the Edit user window.

Create and save files
The Supervisor can check this option to allow the selected user to create and save files. If the user is also allowed to open and view files then this option also allows the user to modify and save existing files.

Open and view files
The Supervisor can check this option to allow the selected user to open and view existing files.

Edit button
Any user can edit private personal information by bringing up the Edit user dialog box. The Supervisor can create and delete other users - not edit them.

Add button
The Supervisor can add a new user by bringing up an empty Edit user dialog box.

Note:
The above options can only be modified by the Supervisor and not by each individual user.
To give full access to a user the Supervisor must check both Create and save files and Open and view files.

The above user setting can only be modified by the Supervisor and not by each individual user.

Delete button
The Supervisor can delete any user account except the Supervisor.

Protocol
The Supervisor can force a fixed protocol/file stamp to be included on every file created (File|New file) by the system. The protocol stamp can only be edited in this menu by the Supervisor and not by normal users of the system. Once this stamp is stored in a file it can never be changed again.

Restrict file access to directory
The Supervisor can check this option to prevent users from opening and saving files outside the specified directory. Sub-folders with-in the specified directory can still be accessed.

Browse button
The Supervisor can click this button to open a browse dialog to select a directory that users will be forced to use as target for files. Sub-folders with-in specified directory can still be accessed.

Lock backup directory settings
The Supervisor can check this option to prevent users from changing the backup directory settings.

Change password every 90 days
The Supervisor can check this option to force users to change their passwords after 90 days.

Lock recovery markers
The Supervisor can check this option to prevent users from making changes to the recovery marker settings.

OK button
Stores all changes and closes the dialog box.

Cancel button
Cancels all operations and closes the dialog box.

Edit user dialog
This dialog box controls the editing of user information.

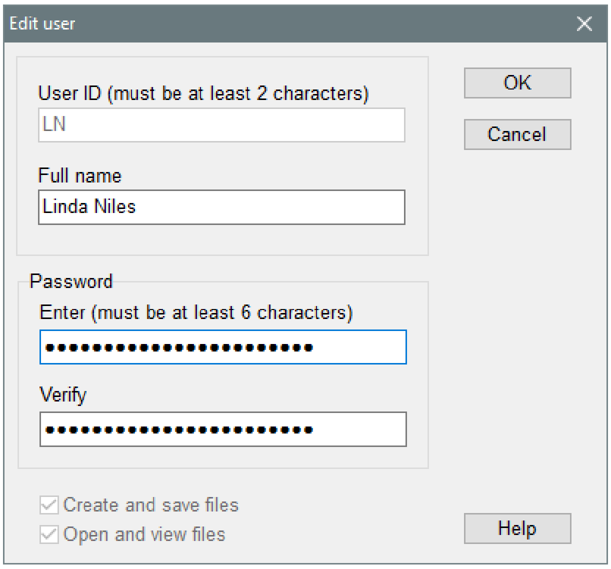

User ID
Add a user ID: Note that the user ID must be unique i.e. no duplicates. Only letters and numbers are allowed in the user ID. The user ID must contain at least two characters. The user ID will be stored in all files and will appear on all print-outs.

Note:
This option can only be controlled by the Supervisor and not by each individual user.

Full name
Type the full name of the user. The full name will be stored in all files and will appear on all print-outs.

Enter Password
Create or change a password. Only letters and numbers are allowed. The password must contain at least 6 characters and are not case sensitive.

Verify Password
Verify the password by typing the above password one more time.

Window Menu

Command	Description
Tile horizontal	Choose Window|Tile horizontal to tile open File Windows horizontally. This option tries to do a horizontal split of open windows so they cover the entire desktop without overlapping.
Tile vertical	Choose Window|Tile vertical to tile open File Windows vertically. This option tries to do a vertical split of open windows so they cover the entire desktop without overlapping.
Cascade	Choose Window|Cascade to stack all open File Windows. This option overlaps each window so they are the same size and only the title bar of each underlying window is visible.
Arrange Icons	Select Window|Arrange Icons to rearrange the icons. Arranged icons are evenly spaced, beginning at the lower left corner of the desktop. At least one File Window must be minimized or this command is useless.
Close All	Close all open File Windows.

Note:
Every open file will also be listed in this menu and clicking on a file name will bring the related file in front.

Help Menu

Command	Keyboard	Description
Help	F1	Help on current topic.
About		Displays the introduction window including the software version of this program.

Pop-Up Menu

By right-clicking anywhere in the Info window or on a grey selection box in the graphic window a small Pop-Up menu will appear.

The Pop-Up menu creates a convenient short-cut to existing menu functions.

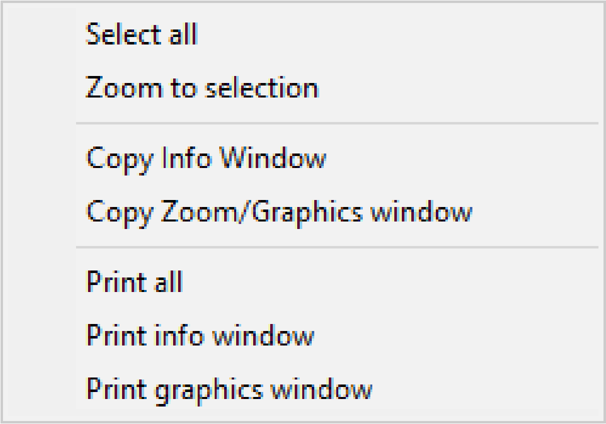

Glossary

ASA Physical Status Classification System

None: A non classified patient.

ASA 1: A normal healthy patient.

ASA 2: A patient with mild systemic disease.

ASA 3: A patient with severe systemic disease.

ASA 4: A patient with severe systemic disease that is a constant thread to life.

ASA 5: A moribund patient who is not expected to survive without the operation.

ASA 6: A declared brain-dead patient whose organs are being removed for donor purposes.

Twitch Cursor
The vertical line pointing from the top border line of the Graphics Window or the Info Window down to the top of a twitch.

Normal Mode
In Normal Mode, only twitches are shown.

Trend Mode
In Trend Mode, all twitches are shown, but for TOF and TOFs stimulations also the calculated TOF result are shown. In case the TOF ratio cannot be calculated a purple block in the bottom of the graph represents the number of detected TOF counts (twitches >= 3%).

Index

Edit menu;31
Environment options;33
Export file;23
File menu;14
File window;9
File window options;35
File|File info;24
File|Logout;30
File|Print graphics window;29
File|Print info window;28
General information;3
Getting started;4
Glossary;49
Graphics window setup;38
Help menu;48
Info window setup;36
Legend window;40
New file;15
Open file;17
Options menu;32
Options|Status Window;41
Pop-Up Menu;48
Save file;20
Search menu;31
Standard Comments;43
Users;46
Window menu;48
